# Mycoplasmas as Host Pantropic and Specific Pathogens: Clinical Implications, Gene Transfer, Virulence Factors, and Future Perspectives

**DOI:** 10.3389/fcimb.2022.855731

**Published:** 2022-05-13

**Authors:** Ali Dawood, Samah Attia Algharib, Gang Zhao, Tingting Zhu, Mingpu Qi, Kong Delai, Zhiyu Hao, Marawan A. Marawan, Ihsanullah Shirani, Aizhen Guo

**Affiliations:** ^1^The State Key Laboratory of Agricultural Microbiology, (HZAU), Wuhan, China; ^2^College of Veterinary Medicine, Huazhong Agricultural University, Wuhan, China; ^3^Department of Medicine and Infectious Diseases, Faculty of Veterinary Medicine, University of Sadat City, Sadat City, Egypt; ^4^Hubei Hongshan Laboratory, Wuhan, China; ^5^National Reference Laboratory of Veterinary Drug Residues (HZAU) and MAO Key Laboratory for Detection of Veterinary Drug Residues, HZAU, Wuhan, China; ^6^Department of Clinical Pathology, Faculty of Veterinary Medicine, Benha University, Toukh, Egypt; ^7^Hubei International Scientific and Technological Cooperation Base of Veterinary Epidemiology, Huazhong Agricultural University, Wuhan, China; ^8^Infectious Diseases, Faculty of Veterinary Medicine, Benha University, Toukh, Egypt; ^9^Para-Clinic Department, Faculty of Veterinary Medicine, Jalalabad, Afghanistan

**Keywords:** mycoplasmas, pantropic pathogens, virulence factors, clinical implications, gene transfer, vaccination

## Abstract

Mycoplasmas as economically important and pantropic pathogens can cause similar clinical diseases in different hosts by eluding host defense and establishing their niches despite their limited metabolic capacities. Besides, enormous undiscovered virulence has a fundamental role in the pathogenesis of pathogenic mycoplasmas. On the other hand, they are host-specific pathogens with some highly pathogenic members that can colonize a vast number of habitats. Reshuffling mycoplasmas genetic information and evolving rapidly is a way to avoid their host’s immune system. However, currently, only a few control measures exist against some mycoplasmosis which are far from satisfaction. This review aimed to provide an updated insight into the state of mycoplasmas as pathogens by summarizing and analyzing the comprehensive progress, current challenge, and future perspectives of mycoplasmas. It covers clinical implications of mycoplasmas in humans and domestic and wild animals, virulence-related factors, the process of gene transfer and its crucial prospects, the current application and future perspectives of nanotechnology for diagnosing and curing mycoplasmosis, *Mycoplasma* vaccination, and protective immunity. Several questions remain unanswered and are recommended to pay close attention to. The findings would be helpful to develop new strategies for basic and applied research on mycoplasmas and facilitate the control of mycoplasmosis for humans and various species of animals.

## Introduction

Mycoplasmas are the smallest and simplest self-replicating microorganisms. Numerous species occur as opportunistic pathogens in mammals, birds, reptiles, insects, and plants (Razin, 1992). Due to their limited metabolic capacity, which is a consequence of their small genome and lack of cell wall, they are fastidious and some of them are laborious to be cultured. As such, they can be both extracellular and intracellular pathogens whose lives depend on the largesse of their hosts (Morowitz and Tourtellotte, 1962).

Mycoplasmas are pantropic *in vivo*. Their favorable localizations are the mucous surface of respiratory and urogenital tracts, mammary glands, eyes, alimentary canal, and joints (Razin et al., 1998). Some *Mycoplasma* species (*M. penetrans, M. pneumoniae, M. fermentans, M. hominis*, and *M. gallisepticum* (*MG*) can adhere to and invade the targeted cells by interacting with their membranes (Baseman et al., 1995; Shibata et al., 2000; Vogl et al., 2008). When two *Mycoplasma* species colonize one habitat, the horizontal gene transfer (HGT) might occur leading to the virulence evolution of mycoplasmas which has a crucial impact on their pathogenesis (Bürki et al., 2015). The emergence of multidrug resistance (MDR) caused by the transfer and/or exchange of antibiotic resistance genes (ARGs) between different pathogens is a growing concern (Forsberg et al., 2012; Faucher et al., 2019). Vaccines are developed and commercially applied in the control of some *Mycoplasma*-related diseases but the efficacy is far from satisfaction. Therefore, novel therapeutic and preventive products are urgently needed to secure animal health, hence improving human health (Fair and Tor, 2014; Valentine-King et al., 2020).

Precisely, perceiving the comprehensive progress, current challenge, and future perspectives of mycoplasmas are helpful to settle down future plans and strategies of basic and applied research on mycoplasmas. Intriguingly, these strategies will facilitate the prevention and treatment of mycoplasmosis for various species of hosts. Therefore, this review aimed to provide an updated insight into the state of mycoplasmas focusing on them as pathogens of human and terrestrial animals. The findings would be applied to get rid of or avoid the growing threat of mycoplasmosis to the health of all affected creatures.

## Clinical Implications of Mycoplasmas

Mycoplasmas are potential pathogenic organisms of humans and many animal species. Pathogenic mycoplasmas have a natural tendency to colonize certain sites *in vivo* called “tissue tropism”, such as respiratory, ocular and genital mucosa, and mammary glands (Razin et al., 1998)

### Human Infection

Mainly, six *Mycoplasma* species (*M. pneumoniae, M. genitalium, Ureaplasma (U) urealyticum, U. parvum, M. hominis, M. penetrans*) have been demonstrated to cause human illness (Waites et al., 2005), such as acute respiratory disease (Waites et al., 2017), joint infections (Ali et al., 2021a; Mărginean et al., 2021), genital and urinary tract infections (Peter et al., 2018), and neurological disturbance (D’Alonzo et al., 2018; He et al., 2021a). On the other hand, some species mainly infecting animals like *M. suis*, *M. ovis*, and *M. haemofelis* have been detected in humans and regarded as zoonotic pathogens (Maggi et al., 2013b). These mycoplasmas can establish persistent infections (Yavlovich et al., 2004), alter host cell physiology, modify apoptotic pathways (Chernov et al., 2015), induce the production of inflammatory substances (Benedetti et al., 2020), and result in cellular DNA damage and cancers (Zella et al., 2018). In addition, serious consequences like chronic obstructive pulmonary disease (COPD) (Feng et al., 2021) and infertility may occur (Kusanovic et al., 2020).

Transmission of human *Mycoplasma* infection occurs through human-to-human contact. It mainly infects mucosal surfaces of the respiratory and urogenital tracts. Droplets containing the organism spread the infection from host to host (Waites et al., 2017). *Ureaplasma* species, *M. genitalium*, and *M. hominis* are genitourinary mucosal organisms and the infection can spread through direct sexual contact, *Ureaplasma* species mainly cause urethral and gynecological infections (Cassell et al., 1993; Lanao et al., 2022).

The clinical picture of *M. pneumoniae* has diversely presented from self-limiting to life-threatening disease (Saraya et al., 2014). For instance, it causes community-acquired pneumonia (CAP) in people of any age, especially in children and young adults (Li et al., 2019; Tsai et al., 2021). *M. pneumoniae* symptoms are variable including fever, cough, sore throat, and occasionally, acute exacerbation of asthma. In addition, severe pulmonary sickness including bronchiolitis, pleural effusion, lung abscess, and pulmonary embolism as a consequence of *M. pneumoniae* infection has also been reported (Meyer Sauteur et al., 2016). The culture procedure is a gold standard technique for *M. pneumoniae* diagnosis. On the other hand, it is recommended to use polymerase chain reaction (PCR) for diagnosing acute cases because culture methods require several days for obtaining results. Moreover, PCR is a rapid, sensitive, specific, and commercially available method, and so it is more suitable for mycoplasmas diagnosis in the clinic (Waites et al., 2017).

Mainly, macrolides, fluoroquinolones, and tetracycline are used for the treatment of *M. pneumoniae* infection; macrolides are the most potent antimicrobial agents for the treatment of mycoplasmosis through inhibition of the bacterial protein synthesis (Waites et al., 2017). Recently, because of the broad use of macrolides, macrolide-resistant *M. pneumoniae* (MRMP) has become increasingly prevalent worldwide (Yang, 2019). The macrolides resistance of *M. pneumoniae* has been emerging worldwide. In Taiwan (2010 to 2017), its rate was 15–30% (Yang et al., 2019a). In America and Europe (2008 to 2013), it was <30%. While in other countries and regions (China mainland, Japan, and Korea) it was about 60–90% (Waites et al., 2017). Age is also regarded as a major determinant for MRMP as the detection rate was higher for children aged ≤15 years than adults. In adults, the detection rate was higher in adolescents (16–19 years) than in older age (≥20 years) (Yamazaki and Kenri, 2016). On the other hand, fluoroquinolones and tetracyclines have more severe side effects than macrolides as tetracycline cause enamel hypoplasia and discoloration of the teeth in young children. Despite the detected hazardous effects of fluoroquinolones on joints and muscles of children, they have been successfully used for the treatment of some complicated cases of MRMP strains in young children (Jackson et al., 2016; Ahn et al., 2021).

Owing to the marked increase of *M. pneumoniae* antimicrobial resistance in recent years as well as the previously mentioned side effects of some antimicrobial agents, the development of protective vaccines against this pathogen is a critical requirement (Jiang et al., 2021). Recently, the designing of the next-generation vaccine approach was performed to establish an effective multi-epitope vaccine (MEV) for human protection against *M. pneumoniae* (Mahmood et al., 2021). To date, Mara et al. (2020) have achieved a breakthrough in explaining how the vaccine-enhanced disease (VED) occurs as a result of *M. pneumoniae* vaccination with lipid-associated membrane proteins (LAMPs). Intriguingly, they demonstrated that *M. pneumoniae* lipoproteins lipid moieties are responsible for VED occurrence. In addition, the removal of lipid molecules from LAMPs before vaccination prevents VED and reduces bacterial loads in the case of *M. pneumoniae* infection. Lipoproteins are the main immunogenic and antigenic constituents of the LAMPs fraction, and therefore their lipid moieties significantly reduced LAMP-stimulated TNF-α production which leads to the VED (Mara et al., 2020). These results may be widely applicable for other mycoplasmas in which vaccine-induced disease exacerbation has been described such as *M. bovis* (Bryson et al., 1999) and *Mmm* (Nicholas et al., 2004).

Nowadays, sexually transmitted antigens are of major concern. Nogueira and co-workers have conducted a recent computational study using *in silico* methods as “subtractive genomics and reverse vaccinology” on five strains of *M. genitalium*, a serious sexually transmissible pathogen. The state-of-the-art sequencing technologies with the availability of the required genomic data paved the way for conducting this work that aimed at predicting the potential vaccine targets and drug candidates. A total of 14 novel vaccine candidates and 2 novel drug targets have been obtained which need further experimental validation to ensure their efficacy for the prevention and control of *M. genitalium* infection (Nogueira et al., 2021). More interestingly, *M. genitalium* is resistant to most antibiotics and difficult to be treated and controlled. Also, it causes endometritis, premature birth, and sterility in women and urethritis in men (McGowin and Totten, 2017). Hence, Ali et al. have conducted proteome-wide vaccine targets prioritization for designing an antigenic vaccine candidate against *M. genitalium* infection. MEV has been constructed successfully with further determining of the different physicochemical properties of the vaccine, but this study still needs further experimental validation for the constructed MEV (Ali et al., 2021b).

More recently, *M. hominis* infection was reported to cause bacteremia, pneumonia, and meningitis, but its significance to cause neonatal meningitis remains elusive. Using CSF patient samples, translucent colonies were observed on chocolate agar media, and the microorganism was recognized as *M. hominis* with MALDI-TOF MS. The 16S rRNA gene sequencing was also carried out which showed 99% nucleotide identity to *M. hominis* (Kersin et al., 2020). *M. hominis* is characterized by a very slow growth rate that requires specific growth media and it’s resistant to many antibiotics such as β-lactams, glycopeptides, sulfonamides, and macrolides (Kersin et al., 2020; Ferreira et al., 2022).

#### Mycoplasma Infection and Respiratory Diseases

**Figure 1:**
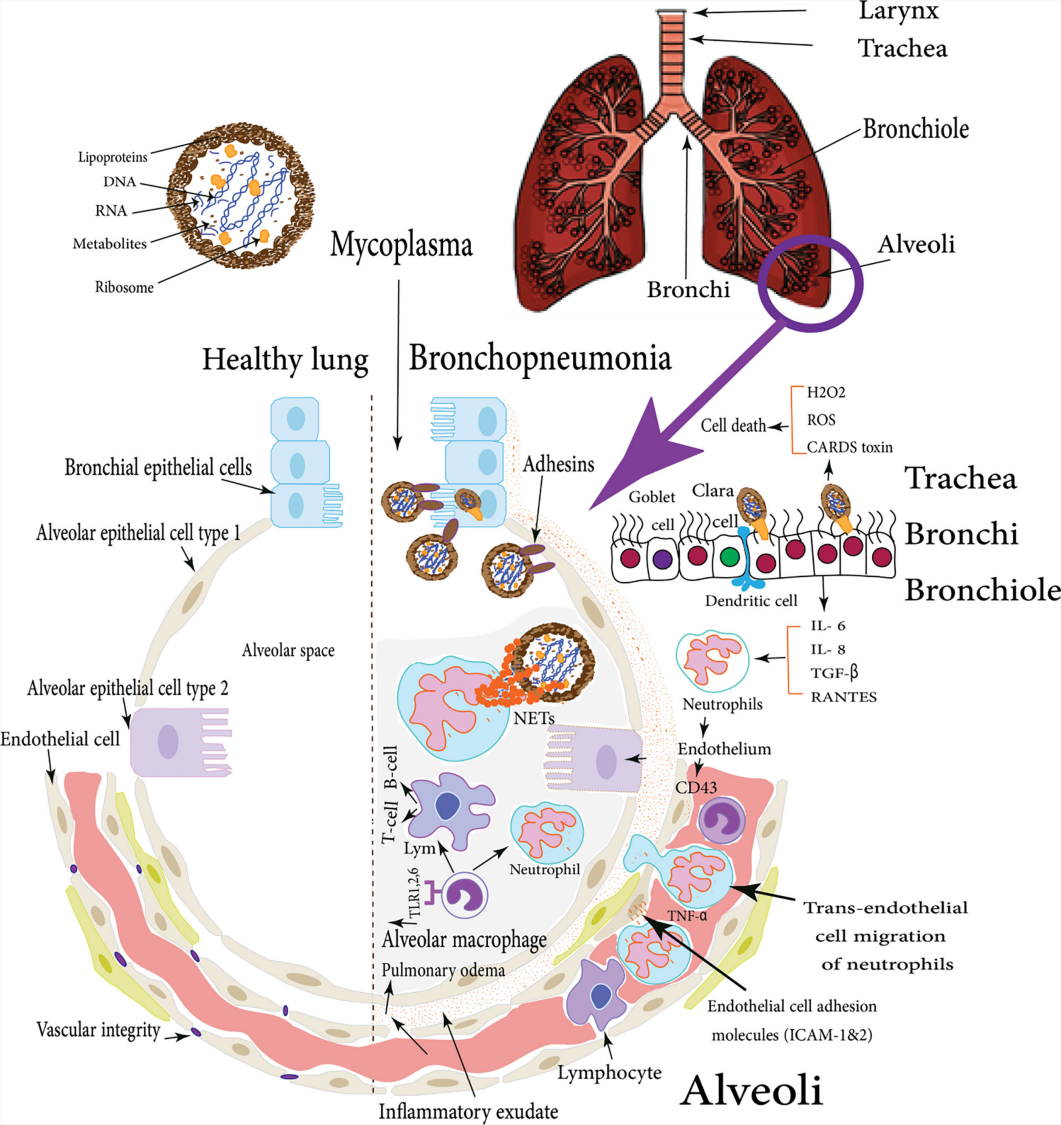
*M. pneumoniae* infection of human lung: the figure illustrates the difference between the healthy lung and *Mycoplasma* bronchopneumonia, the various cellular types incorporated in the respiratory defense mechanisms in case of *M. pneumoniae infection* of human lungs, these cells induce cytokines production which in turn stimulate both types of cellular and humoral immune responses with various virulence factors that enable *Mycoplasma* pathogens to adhere and colonize respiratory epithelial cells (Shimizu et al., 2008).

Traditionally, the clinical picture of *Mycoplasma* infections was more intimately suggestive of damage due to host immune and inflammatory responses rather than direct toxic effects induced by *Mycoplasma* cell components (Razin and Jacpbs, 1992). However, He et al. (2018) have shown that many direct effects, as well as indirect immune mechanisms, have been incorporated in *M. pneumoniae* pathogenesis. The direct effect mechanisms include adhesion damage of *M. pneumoniae* to targeted epithelium then membrane fusion damage *via* alteration in its exposed receptors (Bao et al., 2015). In addition, nutrition depletion is caused by its limited metabolic capacity (Yus et al., 2009). Invasive and toxic damages are following mycoplasmas invasion of different host cells and microbial production of H_2_O_2_ and superoxide. Besides, the produced endogenous toxic oxygen leads to an increase in the intracellular oxygen pressure in the host cells, subsequently; oxidative stress and cell death will occur (He et al., 2018) as shown in **Figure 1**. On the other hand, the indirect immune damage mechanisms include humoral and cell-mediated damages and inflammatory damage *via* an intracellular receptor protein complex (inflammasome) (Shimizu, 2016).

More recently, gene expression analysis and whole transcriptome sequencing have been performed for *M. pneumoniae* infected Hela cells. The results illustrated that protein-coding genes of *M. pneumoniae* are correlated with immune response rather than cellular processes, probably suggesting the intrinsic ability of *M. pneumoniae* to modulate host immune pathways (Ramos et al., 2021).

COPD is one of the foremost predisposing causes of death in the USA, killing > 130,000 individuals per year. Globally, > 3 million deaths annually of COPD (Marciniuk and Schraufnagel, 2017), meanwhile, the middle- and low-income countries are more severely affected. Moreover, the lung microbiota of COPD patients contains more *M. pneumoniae* (Marciniuk and Schraufnagel, 2017). Eventually, *M. pneumoniae* continues to significantly aggravate the onset and recurrence of asthma (Meyer Sauteur et al., 2016).

#### Mycoplasma Infection and Urogenital Diseases

In females, *M. genitalium*, a sexually transmitted pathogen, has been associated with cervicitis, pelvic inflammatory disease (PID), spontaneous abortion, preterm delivery, and infertility. In parallel, it was detected among 10% to 30% as well as 4% to 22% of women with clinical cervicitis and PID, respectively (Gaydos et al., 2009). The high susceptibility of emerging antibiotic resistance is becoming increasingly important (Peter et al., 2018). On the other hand, *M. genitalium* causes symptomatic and asymptomatic urethritis among men and is the etiology of approximately 15%–20% and 40% of Nongonococcal urethritis (NGU) and persistent or recurrent urethritis, respectively (Bachmann et al., 2020).

Additionally, most urinary tract infecting bacteria can be demonstrated on standard culture, but it is exceptional for *Mycoplasma* and *Ureaplasma.* In which, bacterial count in urine does not necessarily relate to the number of bacteria in the bladder wall. A significant number of these intracellular organisms may occur in the bladder wall and be absent in urine. Thereafter, unresponsiveness to antibiotics, persistent lower urinary tract infection, and pyelonephritis were previously reported (Combaz-Söhnchen and Kuhn, 2017). *M. hominis* and *Ureaplasma* infection is notably associated with women’s infertility (Latino et al., 2018; Kusanovic et al., 2020). A recent study has identified these infertility-causing pathogens using the PCR technique. A number of 2360 tissue samples have been collected by urethral and cervical canal scrapings of adult women suffering from PID. The results showed that *Ureaplasma spp.* and *M. hominis* have been identified in 543 and 179 women, respectively. In addition, 112 women had mixed infections (Piscopo et al., 2020).

Furthermore, *M. hominis, U. urealyticum, and U. parvum* are examples of pathogens that can invade pregnant mothers and are closely associated with neonatal pneumonia (Waites et al., 2005). In pregnant mothers, *U. urealyticum* is found in the lower urogenital tract flora, occasionally, it ascends and causes bacterial vaginosis, chorioamnionitis, and premature birth (Stol et al., 2021). In the fetus, it causes neonatal sepsis and meningitis (Ferreira et al., 2021). Both *Ureaplasma spp. and M. hominis* can produce spontaneous abortion with higher rates in the case of *M. hominis* (Latino et al., 2018; Kusanovic et al., 2020). Genital mycoplasmas and ureaplasmas can colonize the urogenital tract which leads to invasive infection and spread to the placenta (Huber et al., 2018). Also, congenital *M. pneumoniae* pneumonia may take place *via* invasion and hematogenous transplacental infection (Hooven and Polin, 2017).

#### Mycoplasma Infection and Joints, Blood, Neurological, and Bone Disorders

*M. pneumoniae* has been frequently involved in severe CNS diseases, such as encephalitis (Al-Zaidy et al., 2015). Besides, it is associated with acute transverse myelitis (ATM) in the form of acute bilateral lower extremity paralysis, paresthesia, and bowel and bladder dysfunction. This syndrome was observed in 15-year-old patients with a slow curing rate that paid attention to the importance of early identification of mycoplasmas infection as a causative agent of ATM and more severe neurological complications (He et al., 2021a). Several extrapulmonary lesions such as cardiovascular, digestive, musculoskeletal, and dermatological lesions during *M. pneumoniae* infection have been summarized in a mini-review reported by (Narita, 2016).

Clinically, arthritis associated with *M. pneumoniae* infection has been diagnosed in children (Azumagawa et al., 2008). In addition, septic arthritis caused by *M. hominis* (Ali et al., 2021a) as well as *U. parvum* (MacKenzie et al., 2010) has been reported in immunosuppressed patients. *M. hominis* has been identified as a novel periprosthetic joint infection (a rare postoperative complication) using a new tool called “metagenomic sequencing” (Wang et al., 2021a). *M. hominis* also might cause brain abscesses (Whitson et al., 2014). What’s more, *Ureaplasma* species have been reported as pathogenic agents causing CNS inflammation in premature babies and abscessation in the adults’ brains (Glaser and Speer, 2015).

Infrequently, *M. orale*, an organism that is generally considered non-pathogenic in humans, has been isolated from patients with immunodeficiency and, as a result, these patients suffered from multiple abscesses and destructive bone disease. For direct detection of the pathogen, surgical specimens were used to do 16S rRNA sequence analysis (Paessler et al. 2002). Recently Ketchersid et al., have described a case report of recurrent multifocal *M. orale* infection in an immunocompromised patient (Ketchersid et al. 2020). Eventually, hemotropic mycoplasmas (HM) are epierythrocytic pathogens that attach to red blood cells of various mammals, including humans causing severe hemolytic anemia (Maggi et al., 2013b; Ikeda et al. 2017).

#### *Mycoplasma* Infection and Cancers

In recent years, many scientists carried out *in vitro* studies using oral tissues (Patil et al., 2015), hepatocytes (Choi et al., 2014) cervical cells (Atallah et al., 2020), and human prostate cells (Abdul-Wahab et al., 2021). These studies concluded that mycoplasmas infection stimulates tumorigenesis by inducing cellular transformation. Yacoub et al. (2021) have demonstrated the possible relationship between mycoplasmas infection and the development of cancers. In parallel, they reported the induction of malignant transformation by mycoplasmas infection in PMNCs (Zhang et al., 2004) and in many other human cell lines such as the uterus SK-UT-1B cells (Polianskaia et al., 1998), A549 lung cells and bone tissues (Jiang et al., 2008), prostate BPH-1 cells (Namiki et al., 2009), and neuronal cell lines (Ji et al., 2019). Another study used the PCR technique to determine that *M. genitalium* levels in patients with prostate cancer were significantly higher than those of patients with benign prostatic hyperplasia (Namiki et al., 2009; Barykova et al., 2011). As such, the available *in vitro* experimental data indicate that *Mycoplasma* infection induces chromosomal alteration, chromosomal instability, and/or cellular transformation *via* genetic mutations and translocations (Paton et al., 1965; Tsai et al., 1995; Feng et al., 1999; Zhang et al., 2000). *M. penetrans, M. fermentans*, and *M. hyorhinis* have been observed to stimulate chromosomal abnormalities, which in turn alter gene expression and cause malignant cell transformation (Benedetti et al., 2020). In addition, *M. hyorhinis* induces hepatocellular carcinoma (HCC) cell migration *via* the interaction of p37 protein with an epithelial cell adhesion molecule (EpCAM). P37 protein plays a key role in facilitating metastases and invasiveness of various cancer cells (Kim et al., 2019). Benedetti et al. have described *Mycoplasma* chaperone DnaK protein as responsible for cellular transformation. Besides they substantiated that this chaperone protein binds to Poly-(ADP-ribose) Polymerase (PARP)-1, a protein that is involved in the repair of any possible DNA damage, and reduces its defensive action. It also binds to USP10 which acts as an essential regulator for p53 protein and minimizes the p53 anti-cancer functions (Benedetti et al., 2020). Furthermore, using the *in vivo* mouse model, it was stated that specific-pathogen-free (SPF) conditions reduced the possibility of tumors formation. Therefore, the diverse microbiome compositions with predominant intracellular mycoplasmas affect the association between the diverse species of *Mycoplasma* and human cancers (Huang et al., 2001; Pehlivan et al., 2004; Pehlivan et al., 2005). In other words, mycoplasmas have been found in many tumor types. So, it is important to identify and characterize the mycoplasmas associated with the tumors in order to determine their role in carcinogenesis (Goodman and Gardner, 2018).

The first report of empyema caused by a commensal human *Mycoplasma* infection was described in a case of right pleural space infection with *M. salivarium* that was accompanied by laryngeal cancer (Baracaldo et al., 2012). *M. salivarium* is commonly responsible for nonpathogenic human infections, but it causes pathogenic infections only in the case of immunosuppressed persons through invasion of the human oropharynx (Totten et al., 2021).

### Animals Infection

Many species of domestic and wild animals suffer from mycoplasmosis. Of which, contagious bovine pleuropneumonia (CBPP) and contagious caprine pleuropneumonia (CCPP) are the two most serious diseases, especially in low and middle-income countries shown by pleuropneumonia accompanied by extremely painful symptoms, reduced productivity, and death. CBPP has higher morbidity compared with CCPP; however, CCPP has higher mortality rates (Bolajoko et al., 2020). Both diseases require accurate diagnostic techniques and improved vaccines which should be accessible in the affected countries (Jores et al., 2020).

#### Bovine Infection

CBPP is mainly a disease of cattle and water buffalo, it is caused by *Mycoplasma mycoides subsp. mycoides* (*Mmm*) and notifiable disease of cattle listed by the World Organization for Animal Health (OIE) (Grieco et al., 2001; OIE, 2021). For the time being, OIE has announced that Europe, the USA, Australia, and South Africa are free from CBPP. For Asian countries, China and India were declared to be officially free, but the disease status is currently unknown in the remaining parts of Asia (OIE, 2019). However, it is endemic in sub-Saharan Africa causing huge annual economic losses (almost 2 billion US$), high mortality (10-70%), severe fibrous bronchopneumonia in the acute cases, and pulmonary sequestra in the chronic stage (Anonymous, 2018).

*Mmm* infection can be summarized in several consecutive stages, firstly, inhalation of infected aerosol droplets; after that, colonization of bronchioles and alveoli, thereby; *Mmm* invades the blood and lymphatic vessels and causes vasculitis. Finally, *Mmm* passes through blood and persists in a variety of other tissues including the lung, in which, the antigen is mainly detected in lung phagocytic cells, on the alveolar and bronchiolar epithelial cells, within the wall of blood and lymphatic vessels, and inside necrotic areas (sequestra formation) as shown in **Figure 3**. Infected animals actively excrete the pathogen through aerosolized droplets as a potential source of infection for the closely in-contact animals (Di Teodoro et al., 2020). The attenuated CBPP vaccine can provide a moderate level of protection estimated by a reduction in lung lesions in vaccinated and challenged cattle. Annual revaccination with the live vaccine is necessary to maintain protective immunity. Additionally, this vaccine is relatively inexpensive and easy to be produced on a large scale (Jores et al., 2020). On the other hand, this vaccine has a short period of immunity with many adverse reactions because it is a live-attenuated type; therefore, its reversion to virulent form sometimes occurs. In addition, it is temperature-sensitive (Dudek et al., 2021). Often, severe inflammation at the injection site followed by skin sloughing has been reported, and so far it can lead to animal death (Jores et al., 2020).

*M. californicum*, *M. leachii*, and *M. dispar* are other mycoplasmas that can cause significant diseases in cattle, but the most important worldwide pathogen infecting cattle is *M. bovis.* It can quickly spread to all age groups. Newborn calves can get the infection from older animals that suffer from severe mastitis, arthritis, and pneumonia (Hazelton et al., 2020) that maintain the infection cycle in the herd. Following a recent survey conducted in the United Kingdom from 2006 to 2017, calves at the age of < 3 months (post-weaning) have the highest prevalence of *M. bovis* pneumonia *Mycoplasma bovis* Investigations in Cattle (2018). Mainly, *M. bovis* infects the upper respiratory tract of young calves during the first few weeks of life through feeding of infected milk and/or direct contact with other infected calves’ nasal secretions (Maunsell et al., 2009). Hence, to stop the infection chain, we must stop the infection spread to the new calves born after the *M. bovis* detection on the farm. Methods for controlling *M. bovis* are culling or isolating *M. bovis* mastitic cows, pasteurization of infected milk, raising the calves separately from older animals, and better milking hygiene and teat dipping. But unfortunately, until now the protective vaccine against this serious pathogen is commercially unavailable (Haapala et al., 2021). Moreover, it is one of the four main bacterial pathogens associated with bovine respiratory disease (BRD) with significant economic losses as a result of higher morbidity and mortality rates, reduced growth performance, and raised costs of prevention and treatment (Kudirkiene et al., 2021).

#### Caprine Infection

CCPP is a fatal contagious illness of goats caused by *Mycoplasma capricolum subspecies capripneumoniae (Mcc)* and a notifiable disease listed by OIE. It has been reported to affect wild and domestic caprines. A recent report has estimated that CCPP has different case fatality rates of 30% in goat herd (n=200) and 8% in sheep flocks (n=400) (Abd-Elrahman et al., 2020) though previous workers have found sheep to be far more resistant (Nicholas et al., 2008). In addition, It is more widely endemic in East Africa, particularly in Kenya, Tanzania, and Ethiopia (Falquet et al., 2014). The first step to establishing a successful vaccine of CCPP is to design a challenge model that can be used to perform essential immunological studies. As this microbe proved to be host and tissue-specific, a novel challenge model has been established following the recent Kenyan outbreak strain ILRI181 in 2012 (Falquet et al., 2014) rather than the old Kenyan strain F38 (MacOwan and Minette, 1976). The base of this model is using two consequent inoculations of aerosols of *Mcc* culture into the nasal cavity of goats than a trans-tracheal inoculation of animals. This model has a morbidity of 100% and a mortality of 50–60% which simulate the natural infection pattern (Liljander et al., 2019). The current CCPP vaccine is a bacterin with saponin adjuvant, scheduled to start vaccination at 4 months of age with revaccination every 6 months. It is expensive due to the fastidious growth requirements of the pathogen and the relatively high total protein required for one dose of the vaccine (Jores et al., 2020).

*M. agalactiae* is a causative agent of an OIE notefiable disease called contagious agalactia (CA) that causes mastitis in dairy goats with formidable financial losses due to arthritis and drop or complete cessation of milk secretion, cachexia, and cornea opacity that can give rise to complete blindness (Santos et al., 2015). In Brazil, the estimated prevalence of CA in goats in different Brazilian provinces such as Rio Grande do Norte, the main goat raising state, was 83.28%, São Paulo was 27.7%, (Azevedo et al., 2015) and Sergipe was 10.3% (Santos et al., 2015; Damasceno et al., 2020). More importantly, many Mediterranean countries are showing substantial losses in the goat dairy industry in France (Poumarat et al., 2016), Spain (Paterna et al., 2013), and Italy (Cillara et al., 2015). CA causes considerable economic losses in Ukraine, according to a recent serological investigation in the Artsyzk area, 168 ewes (32.4 percent) of 519 investigated animals were infected with contagious agalactia. Of which, 109 (64.9%) were in their first year of life, 52 (31.0%) in their second year, and 6 (3.6%) in the 5-6-year-old age group (Bohach et al., 2021).

Hemoplasmas are known as pleomorphic tiny bacteria; they were named because they tend to attach to the erythrocytes’ surface and may cause hemolytic anemia in a wide range of mammals as well. Two well-known *hemoplasmas*, *M. ovis* and *Candidatus M. haemovis*, have been proven to infect small ruminants, their severity increases in young aged and pregnant animals (Hornok et al., 2012). *M. ovis* is a causative agent of chronic infection in caprines. Few reports are available regarding the prevalence of *M. ovis* infection in goats with variable figures ranging from absence in Australia and Tunisia (Rjeibi et al., 2015) to 20% in Hungary and 94% in Malaysia (Jesse et al., 2015).

#### Ovine Infection

Since *M. ovipneumoniae* was isolated for the first time; it is widely known as “sheep atypical pneumonia” specifically infecting sheep and goats (Besser et al., 2013). Latterly, it causes a potential threat to fattening lamb flocks and the lamb industry due to lower lamb growth and decreased ewe productivity rates, it also has been reported in many worldwide epidemics (Bai et al., 2020) (Jaÿ et al., 2020).

Urie et al. had conducted a wide-scale study and estimated the overall prevalence of *M. ovis* infection across the USA, it was 24.3% in domestic sheep (Urie et al., 2019). In another study, *M. ovis* prevalence was high up to 45.8% in 504 sheep samples in China (Wang et al., 2017). Importantly, Maggi and coworkers have reported that *M. ovis*-like species was the most predominant hemotropic organism found in human patients; thus, *M. ovis* could have a zoonotic nature (Maggi et al., 2013b).

#### Swine Infection

*M. hyopneumoniae (M. hyo)* and *M. hyorhinis* (Stemke et al., 1992) have been recognized as the main *Mycoplasma* species that are responsible for various porcine respiratory disorders. Merodio et al. have applied an experimental swine infection model of *M. hyorhinis*, the results indicated that multiple inoculations may simulate subclinical natural infection as in the field. Besides, animals would have to be infected several times for showing a visible immune response (Merodio et al., 2021). *M. hyorhinis* and *M*. *hyosynoviae*, are commensal microbes of the upper respiratory tract and tonsils of swine, they cause arthritis and polyserositis in young pigs between (6-10) weeks of age. While pigs older than 3 months of age are usually suffering from mild arthritis (Neto, 2012). More frequently, *M. hyosynoviae* is known to cause arthritis in adult pigs, but its lesions are restricted to the joints and synovial membranes (Gomes Neto et al., 2015).

*M. hyo* plays a significant role in the development of the porcine respiratory disease complex (PRDC) infection *via* reduced animal growth performance, reduced feed efficiency, and decreased average daily gain. Mostly, an increase in mortality rate takes place with the help of complicated infections (*Pasteurella multocida, Haemophilus parasuis, Streptococcus spp.*, and *Actinomyces pyogenes*) which leads to increased total fatality rates (Olaniyi et al., 2020). Multilocus variable-number tandem repeat analysis (MLVA) and multilocus sequence typing (MLST) are strain typing genetic tools that can be used for *M. hyo* diagnosis (You et al., 2020). A recent study concluded that the most significant histological changes recorded were thickening of alveolar septa caused by neutrophilic cellular infiltration with intraluminal cellular exudate. The majority of pulmonary lesions were chronic (75.81%) (Mucha et al., 2020). Gilts are considered the main source of pathogen inlets because they are mostly exposed to the pathogen during the lactation period (Patterson and Foxcroft, 2019). Vaccination is frequently administrated all over the world with various commercially available *M. hyo* vaccines for not only healthy animals but also infected herds (Maes et al., 2020). For controlling *M. hyo* infection, Sponheim et al., have recommended the deep tracheal catheter as a more sensitive sampling tool, used for *M. hyo* diagnosis, than laryngeal swabs (Sponheim et al., 2020).

Another threat to the pig industry is infectious anemia caused by three *hemoplasma* species*, M. haemosuis*, *M. suis (Eperythrozoon suis)*, and *Eperythrozoon parvum. M. suis* is the main causative agent of swine hemoplasmosis, which in turn adheres to the RBCs surface and triggers their engulfing by the spleen (Petri et al., 2020), as well as causes reproductive failure mainly stillbirths as reported in Southern Brazil (Bordin et al., 2021).

More importantly, *M. suis* has been proved as the first member of the HM group able to invade the erythrocytes of its host. Using electron microscopy, Groebel et al., have discovered a novel *M. suis* invasive strain that causes severe swine anemia with a fatal illness. Such invasion enables it to escape the host’s immune response and antibiotic therapy, and the intracellular lifestyle has clarified the chronic nature of HM infections (Groebel et al., 2009). Moreover, the genus *Eperythrozoon* was previously transferred to the genus *Mycoplasma*. Now it’s classified under a new order called *Mycoplasmoidales* (Gupta et al., 2018).

*M. haemosuis* was associated with fever, anemia, and skin lesions in domestic pigs (Stadler et al., 2020). Genus *Eperythrozoon* has two new blood parasites species (*Eperythrozoon suis* and *Eperythrozoon parvum*), and it was associated with a severe swine disease called “anaplasmosis-like disease” (Splitter, 1950). Globally, the recently detected swine hemoplasmas, such as China (Fu et al., 2017), South Korea (Seo et al., 2019), and Germany (Stadler et al., 2020), have similar clinical signs to those were formerly concluded for *M. suis* infection. Porcine hemoplasmas (PHs) have been detected in the biggest three pork producers worldwide [China (Song et al., 2014), the USA (Guimaraes et al., 2011), and Brazil (Sonalio et al., 2020)], as well as Germany (Normand et al., 2020), France (Brissonnier et al., 2020), Japan (Hornok et al., 2018), and Argentina (USDA, 2020).

#### Avian Infection

Avian mycoplasmosis is caused by four pathogenic mycoplasmas, *MG, M. synoviae (MS), M. meleagradis (MM), and M. iowae (MI).* The *MG* and *MS* are OIE-listed respiratory pathogens that have been causing huge economic losses due to their dramatic drop in egg production, hatchability, weight gain, and feed conversion efficiency. On the other hand, they increase embryo mortality, carcass condemnation, and prophylaxis and treatment costs in layers, broilers, and breeders flocks (Yadav et al., 2021). *MG* is a major Mycoplasma affecting poultry; it causes symptomatic as well as asymptomatic infections. Clinically, it causes chronic respiratory disease in chickens with difficult breathing, sinusitis, airsacculitis, increase embryo mortality in layer parents, and reduce carcass quality in broilers. More seriously, asymptomatic infection also has a formidable impact on the birds as it can be a predisposing factor to more severe secondary bacterial infections. In addition, *MG* may predispose the animal to many viral contagious diseases such as Newcastle disease and infectious bronchitis (Michiels et al., 2016).

More importantly, *MG* has been isolated from many different bird species acting as reservoirs for commercial poultry. For example, it was identified in the tracheal swabs of racing pigeons. However, the examined birds showed unapparent symptoms, they could play a role as the potential carriers of the organism (Tsai and Lee, 2006; Michiels et al., 2016). House finches and other passerines, another free-flying avian species, are regarded as the most serious threat for uncontrollable *MG* infection transmission. Luttrell et al have conducted a field survey for the assessment of *MG* prevalence among these bird species. The testing indicated that 19.1% of 671 birds caught at farms and 11.6% of 387 birds caught at feeder sites had a positive result (Luttrell et al., 2001). *MS* infection sometimes remains asymptomatic, otherwise, it can show signs of lameness, synovitis, mild lower respiratory signs, and airsacculitis (Yadav et al., 2021).

*MG* infection can be transmitted through horizontal and vertical routes, and so prevention and control measures are mainly through biosecurity and vaccination. Live attenuated and/or recombinant live poxvirus vaccines are commercially available against *MG* and *MS* infection. Also, avirulent *MG* live strains (F, ts-11, and 6/85 strains) can be used safely (Yadav et al., 2021). New research proved that 3 consecutive doses of *MG* vaccines, one live followed by two inactivated vaccine doses, provide good protection in layers (Kiers, 2020). In terms of advantages and limitations of *MG* vaccines, the F strain, a field strain with moderate virulence, is preferable in places where wild-type *MG* is highly virulent because it can defeat this virulent *MG* strain. The other *MG* vaccines, ts-11 and 6/85, were used more safely because they were less pathogenic and transmissible toward young progeny. While, they showed a lower potency in field challenge than F strain (US Animal Health Association, 2006). The *MG* 6/85 vaccine strain was developed through serial passages of a field isolate originating from the United States On the other hand, *MG* bacterins are becoming less popular in commercial flocks, where long-term control of *MG* infection is critical issue. Further, bacterins are more expensive and inappropriate as they need individual vaccination of birds (Ishfaq et al., 2020).

The ts-11 strain of *MG* and MS-H strain of *MS* are temperature-sensitive strains; both of them were proved to be safe and effective for protection against challenge in both chickens and turkeys when administered by eye drop. Globally, both of them are commercially available. For instance, its administration in Australia has greatly reduced the prevalence of disease in chickens causing a tenfold reduction in the use of macrolides in poultry (Browning et al., 2011).

The strain ts-11 vaccine, a mutant induced by chemical mutagenesis, can produce long-term immunity in chickens, but the protective immunity obtained by this vaccine is dose-dependent. A strain ts-304 has been isolated from ts-11 and demonstrated to be as safe as the ts-11 strain. Surprisingly, it also has been effective but at a lower dose and protective against challenge with the *MG* wild-type strain. In addition, its protection lasts for at least 57 weeks after a single vaccination at 3 weeks of age (Kulappu Arachchige et al., 2021). Since live vaccines are used in many parts of the world, Sulyok and his team have developed new highly specific molecular methods to rapidly differentiate *MG* vaccine strains from field virulent isolates using clinical samples (Sulyok et al., 2019).

*MI* is primarily infecting turkeys and occasionally chickens. The natural *MI* infection in turkeys results in late embryo mortality, a drop in hatchability, and leg abnormalities in young chicks (Pritchard and Balish, 2015). *MM* is responsible for air sac disease and musculoskeletal and reproductive disorders mainly in turkeys, it also has been isolated from chickens (Béjaoui Khiari et al., 2011).

#### Equine Infection

*Mycoplasma* infection was rarely reported in horses; however, *M. felis* has been isolated from pleuritis and lower respiratory tract infection cases in equines (Wood et al., 1997). In Japan, using genomic DNA for nanopore sequencing, *M. felis* strain Myco-2 has been detected from a tracheal wash sample of a diseased horse that suffered from respiratory manifestations. This strain has 98.2% identical nucleotides to the typical reference feline strain ATCC 23391 (Kinoshita et al., 2020). In addition, *M. equigenitalium (equi)* is a potential cause of infertility, endometritis, and abortion in mares, besides, reduced fertility in stallions (Tortschanoff et al., 2005). It is difficult and time-consuming to identify *M. equi* in clinical samples, and thus, Nehra et al., have developed a species-specific PCR for *M. equi* diagnosis in clinical samples (Nehra et al., 2015). Two unidentified *Mycoplasma* strains (N3 and NI1) isolated from the equine respiratory tract were proven to have cross-reactions with strains of *Mmm* and *M. mycoides subsp. capri (Mmc)* (Lemcke et al., 1981).

*M. equirhinis* was isolated from 10.2% of tracheal wash samples from racehorses in Great Britain (Cardwell et al., 2013) and 16.2% from thoroughbred horses in Turkey (Mete and ÖZGÜR, 2017). More recently, using the loop-mediated isothermal amplification (LAMP) assay, *M. equirhinis* was isolated from 40.0% of Japanese horses (Uchida-Fujii et al., 2021).

Equine hemoplasmas were discovered for the first time in Germany in 2010, as a new species of hemoplasma (Candidatus *M. haemobos*- like species). After that, scientists have recorded their incidence (26.5%) using a novel real-time PCR assay (Dieckmann et al., 2012). The chronically infected animals could act as reservoirs of infection to other in-contact animals; a recent study discovered *M. ovis*-like species in an index horse case. What’s more amazing is that the molecular and phylogenetic analysis of the haemoplasma sequences had 100% identity with 16S rRNA of *M. ovis*, a hemoplasma mainly related to sheep and goats (Kalantari et al., 2020). The previous discovery means that interspecies transmission of *Mycoplasma* infection could occur anytime. Based on the results of R segment analysis, a species of human *Mycoplasma* is a group of strains that share R-segments with average nucleotide identity (ANIs) ≥97%. Moreover, R-segments are superior to 16S rRNA gene sequences and multilocus sequences for the identification and phylogenetic analysis of human Mycoplasma species and their strains (Roachford et al., 2019).

HM infection in horses seems to behave subclinically with low bacterial blood loads as represented by Dieckmann et al. (2012). In addition, the infected horses can act as a potential reservoir of infection by *M. ovis*-like species for both sheep and humans (Kalantari et al., 2020). Further, Manguin et al. have screened the tracheal microbial inhabitants in asthmatic horses with qPCR and determined that *Mycoplasma spp.* were included in the microbiome composition of tracheal mucus in horses and asthmatic children, as well (Manguin et al., 2020).

#### Canine Infection

More than fifteen different *Mycoplasma* species have been isolated from dogs. They are mostly commensal organisms with a few harmful agents. *M. cynos* was significantly associated with lower respiratory tract (LRT) disease in dogs. On the other hand, no significant association was detected between *M. canis, M. spumans, and M. edwardii* and clinical signs of canine LRT disease (Jambhekar et al., 2019). *M. spumans* and *M. maculosum* were identified by PCR and sequencing to be responsible for fertility problems in male and female dogs (Tamiozzo, 2021).

*M. hemocanis (Mhc*) and *Candidatus M. haematoparvum (CMhp)* are two hemoplasmas species that have been reported in canines. In Italy, symptomatic infection by *CMhp* in a dog was firstly reported by Rosanna et al., who recommended PCR as a gold standard technique for clinical diagnosis of this pathogen (Rosanna et al., 2020). In Korea, the index case of *Mhc* infection was reported in a dog showing clinical signs of severe hemolytic anemia (Kim et al., 2020).

*M. cynos* causes upper respiratory disease in dogs, and it is proved to be associated with increased severity of canine respiratory disease complex (CRDC). Clinical signs may include cough and accumulation of mucus and exudate. Potentially, this microbe often evades the immune response predisposing animals to chronic and secondary bacterial infections (Chalker, 2005).

#### Feline Infection

Four species of feline hemoplasmas have been characterized in domesticated cats. They include *M. haemofelis (Mhf)*, Candidatus *M. haematoparvum*-like, Candidatus *M. haemominutum* (*CMhm*), and Candidatus *M. turicensis (CMt).* In China, the first identified feline hemoplasma in cats was Candidatus *M. turicensis* (*CMt)* (Zhang et al., 2021b). In Thailand, another study estimated that 16.1%, 24.5%, and 1.6% of the random samples collected from stray cats were infected with *Mhf*, *CMhm*, and *CMt*, respectively (Kamyingkird et al., 2021). In Russia, the estimated prevalence of *CMhm, Mhf*, and *CMt* was 7.6%, 5.5%, and 0.7%, respectively (Demkin and Kazakov, 2021). *CMhm* is the most common type of *Mycoplasma* species producing hemolytic anemia. *Mhf* causes a more severe and fatal form of hemolytic anemia in cats, whereas *CMhm and CMt* have lower severity, but only cause severe infection in immunocompromised cats (Willi et al., 2006). Feline infectious anemia is a disease condition of cats accompanied by severe anemia upon erythrocyte disruption. It is induced following infestation by infectious agents such as hemoplasmas (previously mentioned) and *Bartonella species* (intracellular vector-transmitted pathogens infecting cats) (Zhang et al., 2021b). The non-hemotropic *Mycoplasma* (*M. felis*) causes different disease lesions in cats including conjunctivitis, respiratory symptoms, and polyarthritis (Greene and Chalker, 2012).

#### Wild Animal Infections

For tortoises, Origgi and Jacobson stated that the most significant bacterial disease that seriously affects the endangered free-ranging and captive tortoises is mycoplasmosis (Origgi and Jacobson, 2000). Mycoplasmas cause upper respiratory tract infection in threatened species, including gopher and desert tortoises in the USA. More specifically, The *M. alligatoris* causes pneumonia, synovitis, and polyserositis in American alligators (Valentine-King et al., 2020). CCPP affects various species of ungulates, chiefly wildlife species, such as gazelles and some species of antelope-like gerenuks (Baziki et al., 2020). *M. ovipneumoniae*, another *Mycoplasma* species, causes pneumonia in wild caprines (Jaÿ et al., 2020). This pathogen possesses LAMPs that are considered to be the most potent stimulator of inflammatory cascades (Bai et al., 2020).

More disturbingly, many recent studies have emphasized the importance of cervids as reservoirs for mycoplasmas infection since these species are considered the essential food source for many predators (Petri et al., 2020). Boes et al. have demonstrated the first natural HM infection in white-tailed deer. Following the high identity of 16S rRNA to the previously described *M. ovis* organism (Messick et al., 1998), the hemoplasma detected in his study likely represents a strain variation of *M. ovis*, an erythrocytic parasite of ovines (Boes et al., 2012). André et al. have molecularly detected HM in wild canids for the first time in Brazil that are regarded as endangered species; therefore studies concerning their pathogenic threats to their health are critically concerned (André et al., 2011). *M. ovis* is a zoonotic pathogen that has already been demonstrated in reindeer (Stoffregen et al., 2006) and white-tailed deer species (Maggi et al., 2013a) in captivity in the USA as well as in free-ranging spotted deer species in Japan (Watanabe et al., 2010). In Brazil, *M. ovis* has been detected in free-ranging marsh deer and pampas deer species (Grazziotin et al., 2011). A recent study identified for the first time, the occurrence of *M. ovis* in the gray brocket deer and small red brocket deer in the Brazilian national conservation plan for endangered South American deer (André et al., 2020).

*M. conjunctivae*, an important contagion of wild caprinae, cause infectious keratoconjunctivitis (IKC) in the form of mild symptoms in domestic sheep and goats, while it provokes a severe inflammation of conjunctivae and cornea in wild caprinae. It was responsible for severe epidemics episodes in wild caprinae including chamois and ibex (Marco et al., 2009). In the most advanced stages of IKC, corneal ulceration and perforation, as well as 30% mortalities, have been reported. Eye blindness is a consequence of bilateral eye infection which increases the fatality rate, especially in steep rocky areas because of the falling of affected animals from cliffs (Giacometti et al., 2002).

In view of the increasing transmission of *MG* to house finches in the wild, and alarmingly, it was responsible for the death of over 200 million birds (Nolan et al., 1998), this marked the first epidemic of *MG* in the wild birds. Besides, the excessive speed at which this pathogen goes rampant among the house finch population illustrates the rapid pathogen dissemination throughout a large geographic area within a very gregarious and mobile host population (Fischer et al., 1997). Till now, *MG* has been expanding its host range. For instance, it was identified in many phylogenetically different birds including songbirds (Fischer et al., 1997), raptors (Wrobel et al., 2016), and wild passerines (Luttrell et al., 2001). Hence, rapid evolutionary changes of the pathogen as it expanded geographically allow it to be one of the most recognized wildlife pathogen outbreaks (Sawicka et al., 2020). A possible explanation of the aforementioned host diversity or switching might be due to a shift in CRISPR system dynamics. Also, the gradual degradation and critical functional loss of the CRISPR system in house finches *MG* after the host switch appears to have a great impact on the pathogen evolution (Delaney et al., 2012). More recently, *Mycoplasma* infections have been found in migratory wild geese, while, the question concerning the pathogens’ transmission and dispersion is still poorly understood (Sawicka-Durkalec et al., 2022)

#### Laboratory Animals

##### Laboratory Animals’ Infection

Laboratory animals are useful fundamental scientific tools; the progression of apparent or in-apparent infections with *Mycoplasmas* has tremendous alterations on the normal physiological responses of mice throughout experiments. When mycoplasmas are running rampant in the experimental animals’ population, the only solution will be through introducing specified pathogen-free (SPF) animals and animal facilities to avoid potential false results. *M. collis*, *M. pulmonis*, *M. neurolyticum*, *M. muris*, and *M. arthritidis* are the most common *Mycoplasma spp.* infecting mice (Masoumalinejad et al., 2018).

*M. pulmonis* is the most prevalent *Mycoplasma* pathogen in mice causing otitis media, reproductive disorders, as well as substantial respiratory consequences with a prevalence of (20-60) % (Booth et al., 2014). The most important issue regarding *M. pulmonis* infection in rodents is that it is probably the best model from which we have learned the most about the determinants of immunity to control human *M. pneumoniae* respiratory infections. Many studies have used the experimental *M. pulmonis* rat infection model as an ideal model in terms of the ciliary cell function and cellular kinetics (Lambert et al., 1998), neurogenic inflammation (McDonald et al., 1991), natural killer cell activity (Kamiyama et al., 1991), local and systemic immune response (Steffen and Ebersole, 1992), induction of the production of several cytokines (Faulkner et al., 1995), and polyclonal proliferation of B and T lymphocytes (Rocha Sobrinho et al., 2011). *M. neurolyticum* has been characterized as a mammalian brain organism responsible for nerve disorders as a result of secreted *Mycoplasma* toxins (Tully, 1981). Also, *M. collis* was isolated for the first time from the conjunctiva and nasal cavity of mice and rats (HILL, 1983). *M. muris*, a scarce *Mycoplasma* type causing a huge hazardous effect on the reproductive efficiency of female mice, has been identified in recent years (Zinatizadeh et al., 2017). *M. arthritidis*, another rare pathogen of mice, is regarded as the main cause of arthritis in mice with swelling of legs and fingers (Constantopoulos and McGarrity, 1987).

##### Animal Models for *Mycoplasma* Infection

The laboratory mice are the most common species used in animal experimentation in biomedical research. In addition, the experimental mouse mastitis model allows us to examine a large number of *Mycoplasma* strains (Dmochowski, 1967).

Saraya et al., have designed five mouse models for *M. pneumoniae* pneumonia to examine the pathological picture in animals with various immune statuses. Firstly, animals were immunized following different regimes (one for each animal model). Afterward, they were challenged with *M. pneumoniae* antigen intratracheally, only mice groups immunized with *M. pneumoniae* antigen and alum adjuvant or *M. pneumoniae* antigen with CpG adjuvant (Th2 predominant) have developed severe lymphoplasmacytic infiltration in the peri-bronchovascular areas (PBVAs). These results indicate that the adaptive host immune responses in these two models seem to be the main regulator for human *M. pneumoniae* pneumonia pathological features and Th2 predominant characteristics might be important to generate and simulate the typical picture of *M. pneumoniae* pneumonia (Saraya et al., 2014).

*In vivo* strategies whereby BALB/c mice were injected subcutaneously with the T-B epitope peptides resulted in strong antigen-specific serum antibody and cellular immune responses, besides decreasing the inflammatory response of the challenged mice with *M. pneumoniae* (Weng et al., 2017), we can take the advantage of these findings for *Mycoplasma* vaccine production.

Several examples of useful *Mycoplasma* animal models including gerbils (burrowing mouse-like rodents) inoculated intranasally with *M. pneumoniae* to investigate its pathogenesis of human lung infection (Rodríguez et al., 2021). A rabbit model was used in previous studies for the development of polyclonal and monoclonal antibodies against various human diseases (Kaur et al., 1998). Furthermore, hamsters were injected intratracheally with *M. fermentans* culture to explain the ability of the pathogen to induce pneumonia and chronic infectious diseases in humans (Yáñez et al., 2013).

Guinea pigs are regarded as the best animal model after non-human primates to study *M. pneumoniae* infections. In an important study, Dumke et al. have used these animals for studying the pathogen-host relationship as well as characterization and subtyping of *M. pneumoniae* strains isolated from human patients. The adaptation, preference, and survival of individual strains also have been investigated. They concluded that *M. pneumoniae* species is genetically highly homogeneous (Dumke et al., 2004). Hausner and his team have also immunized guinea pigs with a hybrid protein composed of adherence-related parts of the proteins P1 and P30 of *M. pneumoniae*. The results showed a dramatic decrease in its detection in pulmonary samples from vaccinated as well as subsequently infected animals (Hausner et al., 2013). Meanwhile, sera from immunized animals have been demonstrated to have crucial adherence-blocking properties. Besides, the initiation of potent stimulation of mucosal immunity was the milestone for successful vaccination with intranasal antigen as well as in combination with other biocompatible adjuvants (Zhu et al., 2012).

*In vivo* studies using non-human primates have also played a vital role to investigate the antigenic and immunogenic properties as well as the pathogenicity of specific mycoplasmas including *M. geni*talium. For instance, experimentally infected primates have been used to examine *M. genitalium* membrane topology, antibody accessibility, amino acid diversity, and the location of functional and antigenic epitopes for the MgpB adhesion (Iverson-Cabral et al., 2015). Another important example of using primates instead of humans for doing essential experimental work is macaque (a genus of Asian monkeys) which was used for studying the persistence, immune response, and antigenic variation of *M. genitalium* in an animal experimental infection model (Wood et al., 2013).

Large animal models were also established for the *Mycoplasma* study. The experimental infection of SPF lambs with *M. ovipneumoniae* resulted in the establishment of asymptomatically infected upper airways in absence of other secondary infections (Davies et al., 1981). *M. bovis* calf infection model was used to estimate the effectiveness of some antimicrobial agents against animal mycoplasmas (Dudek et al., 2019). These previously illustrated experimental models to study mycoplasmas are valuable in many points of view such as investigation of their infection pathogenesis and immune response, assisting in the development of therapeutic strategies and diagnostic biomarkers, and conducting the potential vaccine candidate’s trials. On the other side, some encountered limitations should be taken into account as the availability of SPF conditions to avoid false results.

## Virulence-Related Factors of Mycoplasmas

The poor understanding of the pathogenesis and immune response for the genus *Mycoplasma* is the main restraint that hampers mycoplasmas diagnosis, prevention, and treatment. Since the shortage of effective genetic tools, late publicized genome sequences, and lack of small animal models, the discovery of virulence factors has been progressing very slowly. Generally speaking, the following virulence-related factors have been considered, including adhesion and invasion, activation of some critical molecules and pathways related to innate and acquired immunity, phenotype variation such as phase variation and antigen shift; generation of secondary metabolites such as hydrogen peroxide (H_2_O_2_), biofilm formation, etc “and so on”. Lipoproteins and secreted proteins of mycoplasmas are important components inducing these activities. The characterization of these proteins might help elucidate pathogenesis and immune response, identify novel target biomarkers, establish diagnostic methods, and make improved vaccines (Zubair et al., 2020b).

### Adhesion and Host Immune Response

Adhesion is the first step of *Mycoplasma* infection. Because it doesn’t have a cell wall, the adhesion is mainly mediated by cellular membrane proteins. For *M. pneumoniae* infection, it firstly attaches to ciliated respiratory epithelial cells at the base of the cilia employing a complex terminal organelle at one end of the elongated organism. Adhesion is mediated by interactive adhesin (P1) (Razin and Jacpbs, 1992) that is translocated to the surface and localized correctly within the attachment organelle. It also maintains interactive stability with accessory structural high molecular weight proteins 1 (HMW1), 2 (HMW2), 4 (HMW4), 5 (HMW5), P90, and P65 clustered at the tip of the organelle (Widjaja et al., 2020) (Waites and Talkington, 2004). Next, *M. pneumoniae* produces hydrogen peroxide (H_2_O_2_) and superoxide radicals (Shimizu, 2016), which induce oxidative stress in the respiratory epithelium. It was reported that *M. pneumoniae* induces transforming growth factor beta-1 (TGF)-β1 in primary cultures of normal human bronchial epithelial cells and RANTES in small airway epithelial cells (Dakhama et al., 2003). Similarly, it would act *in vivo* by inducing TGF-β1 in large airways and RANTES in small airways together with increased IL-6 and IL-8 production on bronchial epithelial cells. On the other hand, neutrophils, the first line of body defense mechanism, secrete chemotactic signals that attract monocytes, dendritic cells (DCs), and macrophages. They produce tumor necrosis factor-alpha (TNF-α) which drives DC and macrophage differentiation and activation (Wang et al., 2018). More intriguingly, some studies have identified that *M. pneumoniae* surface lipoproteins can trigger Toll-like Receptor (TLR) activation, leading to the production of IL-6 pro-inflammatory cytokines. These cytokines activate the transcription factor NF-κB, which translocates to the nucleus to express pro-inflammatory genes which in turn provoke inflammation and cellular immune response (Segovia et al., 2018).

They also directly activate DCs *via* cell-to-cell contact through neutrophil CD11b. Afterward; neutrophils are activated to release small amounts of elastase, which induces endothelial cells to secrete molecules like CD43 allowing closer interaction and stronger binding. Then, neutrophil adhesion is facilitated by the up-regulation of pro-inflammatory cytokine (TNF-α) and endothelial cell adhesion molecules (ICAM-1&2), after that, trans-endothelial cell migration of neutrophils takes place. Yamamoto et al. found that *M. pneumoniae* releases a secreted protein nuclease Mpn491 that can escape neutrophil extracellular traps (NETs)-degrading the ability of neutrophils (**Figure 1**) (Yamamoto et al., 2017). In the absence of this enzymatic activity, NETs can be induced and the networks of extracellular molecules bind *M. pneumoniae* enabling neutrophils to destroy the extracellular pathogen and minimize the disturbance of their host cells (**Figure 1**) (Zhao et al., 2021b). Moreover, *Mycoplasma* lipoproteins induce TLR2 signaling that induces neutrophil NETosis. Remarkably, (NETs)-degrading ability diminishes for older ages, and thus, older patients are more vulnerable to mycoplasmas infection like *M. pneumoniae* (Xu et al., 2017; Christodoulides et al., 2018). Finally, inside the alveoli, if *M. pneumoniae* gets rid of NETs, it will attach to alveolar macrophages (AMs). Subsequently, it is recognized *via* TLR1, 2, and 6 on AMs which originate from blood monocytes which constitute approximately 93% of the pulmonary macrophage population and are the early effectors of innate immunity against any bacteria (Shimizu, 2016).

A cytoadhesion assay was developed to measure the interaction of *Mmm* with different host cells. The results indicated that *Mmm* cytoadherence is tissue and host-specific. In this study, the *in vitro* inhibitory effect of *Mmm* monoclonal antibodies (mAbs) against *Mmm* adherence to bovine lung epithelial cells (BoLEC) was investigated. Aye and coworkers demonstrated that 13 anti-*Mycoplasma mycoides* subspecies *mycoides* (AMMY) mAbs inhibited adhesion by at least 30%. More specifically, AMMY 10, a capsular polysaccharide (CPS) specific antibody, inhibited the *in vitro* growth of *Mmm.* Also, polyclonal rabbit serum against recombinant MSC_0267 blocked the adhesion of *Mmm* to BoLEC by 41%. Further *in vivo* studies are required for exploring the immune response induced by *Mmm* antigens recognized by these antibodies (Aye et al., 2018). A precision-cut lung slices (PCLS) infection model for *Mmm* has been established to study host-pathogen interactions. Using immunohistological analysis (IHA) and electron microscopy, the results of this *ex-vivo* infection model mimic the *in vivo* situation. It showed a consistent increase in the number of adherent *Mmm* Afadé in the bovine PCLS than caprine PCLS over time. Conversely, the adherent *Mmc* was not strongly affected by the type of host tissue as we observed an increase in caprine and bovine PCLS. *Mmc* displayed higher tropism to sub-bronchiolar tissue in caprine PCLS. Furthermore, *Mmc* was abundant on pulmonary endothelial cells which indicates how it causes systemic disease (Weldearegay et al., 2019).

**Figure 2:**
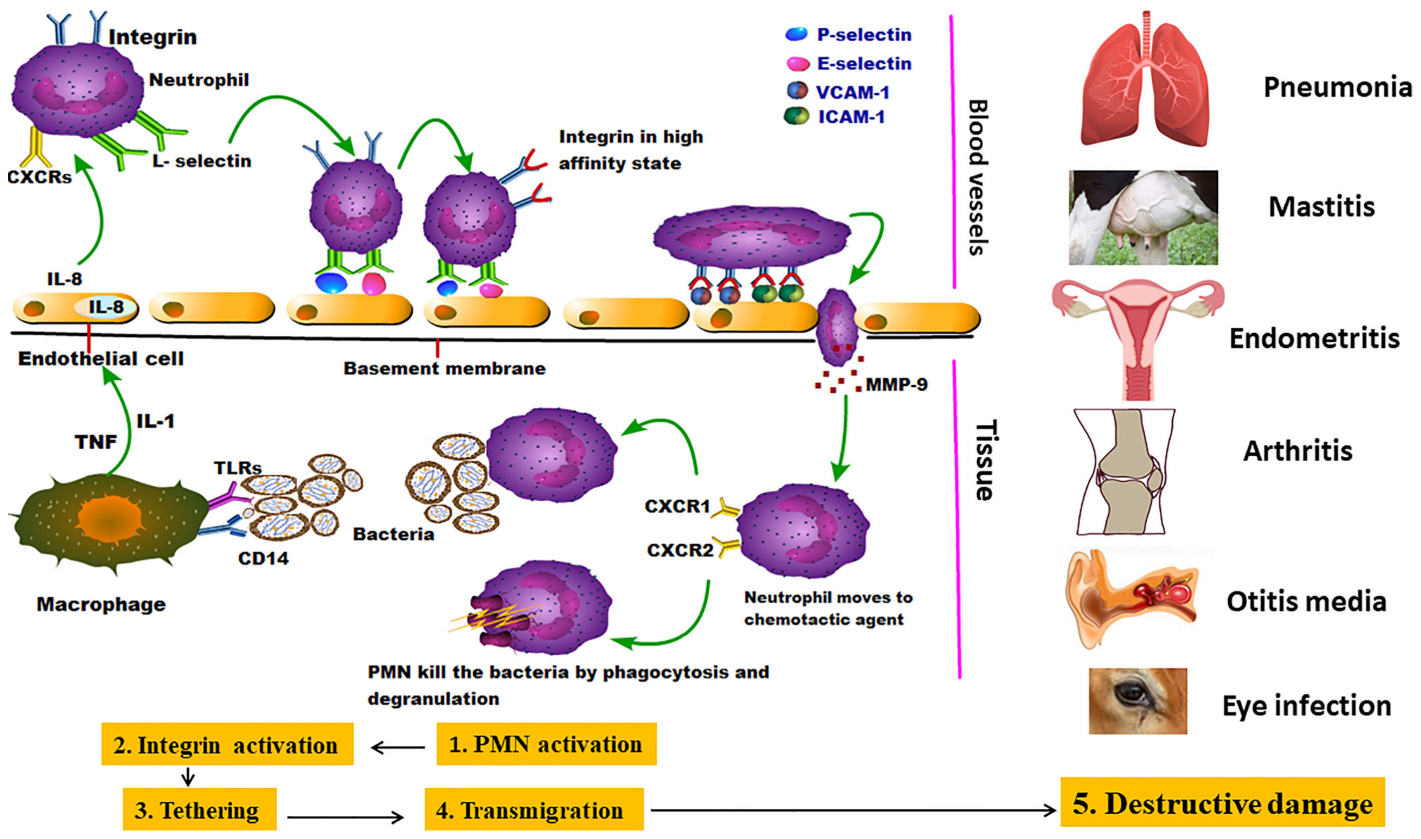
Invasion of mycoplasmas to target tissues and their potential interaction with immune cells: the figure showed the pantropic nature of mycoplasmas starting by stimulating the first line of immune cells (neutrophils) which in turn emit different danger signals and initialize the subsequent stages of PMNCs activation, Integrin activation, Tethering, Transmigration, and Destructive damage in many parts of the body causing different inflammatory lesions (Leick et al., 2014).

The adhesion of animal mycoplasmas to host cells might be started by up-regulating the expression of endothelial cell P-selectin (CD62), E-selectin, vascular cell adhesion molecule-1 (VCAM-1), and intercellular adhesion molecule-1 (ICAM-1). After that, mycoplasmas attach to neutrophil L-selectin (CD62L). The second stage is presented by neutrophil activation to release elastase, which cleaves anti-adhesive molecules (CD43) from the endothelial cells, leading to stronger integrin-binding allowing the neutrophils to transiently attach to the endothelial cells as they pass along. Other well-documented adhesins were mentioned in detail (**Table 1**). The third stage (tethering) slows down the neutrophils, allowing them to interact more with the vascular endothelial cells (Granger and Senchenkova, 2010). *M. pneumoniae* cytadherence initiates inflammatory responses *via* an intracellular receptor protein complex called the inflammasome (Shimizu, 2016). Subsequently, extravasation and migration of neutrophils into the airways take place as a pivotal process to fight the bacterial infection. Through the endothelial cell layer and basement membrane, neutrophils attach the intercellular adhesion molecules (ICAM-1 and ICAM-2) within endothelial cell tight junctions (Chong et al., 2021). Eventually, Matrix Metalloproteases (MMPs) remodel the extracellular matrix to increase cell migration easily through tissues and move toward chemotactic agents (**Figure 2**) (Leick et al., 2014). Major Band Antigen (MBA), a surface-exposed lipoprotein, is a major determinant in the pathogenesis and virulence of the *Ureaplasma* species for causing chorioamnionitis. The potential pathogenesis for this pathogen perhaps caused by some antigenic variations of MBA leads to ureaplasmas escaping the host immune system, and colonization of the upper urogenital tract (Sweeney et al., 2017).

**Table 1:** Overview of virulence-related factors of *Mycoplasma* species at the protein level.

Virulence factors		*M.* species	Significant functions		Reference
CPS	PdhA, pyruvate dehydrogenase (lipoamide) alpha chain (MSC_0265)	*Mmm* *M. pneumoniae*	Immunogenic with diagnostic potential Essential for adhesion, cell invasion, phase variation, and defense against immune systems such as antiphagocytosis and anti-bacteriolytic activity		(Jores et al., 2009)
	PdhB, pyruvate dehydrogenase (lipoamide) beta chain (MSC_0266)				(Naseem et al., 2010)
	PdhC, oxo acid dehydrogenase acyltransferase (Catalytic domain) (MSC_0267),				(Krasteva et al., 2014)
	PdhD, dihydrolipoamide dehydrogenase (MSC_0268).	*Mmm*			(Jores et al., 2009)
	pyruvate dehydrogenase E1a subunit	*M. pneumoniae*	Mediate adhesion to fibronectin Essential as a structural protein for the assembly and/or regulation of cytoadhesion-associated proteins		(Liu et al., 2012b)
	CPS	*M. ovipneumoniae*	Has a cytotoxic effect and induces apoptosis of sheep airway epithelial cells through a ROS-dependent JNK/P38 MAPK mechanism		(Jiang et al., 2017)
	Exopolysaccharide -I (EPS-I)	*M. pulmonis*	Facilitating the dissemination and sustaining a chronic infection through antiphagocytosis or downregulating the functions of macrophages		(Shaw et al., 2013)
Nicotinamide Adenine Dinucleotide (NADH) oxidase (NOX)		*M. bovis*	NADH oxidizing and O2 reducing enzyme. Besides, adherence to embryonic bovine lung (EBL) cells		(Zhao et al., 2017)
Extracellular DNA (eDNA)			Important nutrient for mycoplasmas proliferation in cell culture conditions Its supplementation was accompanied with cytotoxicity for actively dividing host cells		(Zhu et al., 2019)
(rMbovP327) (rMbovP328) (rMbovP276)			NanoRNAs degradation It exhibited activity towards cyclic dinucleotides and nanoRNAs A member of the membrane-associated phosphodiesterases that participates in cyclic dinucleotide and nanoRNA degradation		(Zhu et al., 2020a)
MbovP579			A novel rMbovP579-based ELISA is a highly sensitive and specific method for the early diagnosis of *M. bovis* infection because it acts as a sensitive and specific antigen for the detection of antibodies in sera from both infected and vaccinated cattle		(Khan et al., 2016)
MbovP730			It is a sensitive and specific antigen for the differentiation of infected and vaccinated animals (DIVA) assay It’s based iELISA was established. For clinical samples, this ELISA provided a sensitivity of 95.7% and specificity was 97.8%		(Khan et al., 2018)
MbovP274 MbovP570			Secretory and highly immunogenic proteins that significantly increase the production of IL-8, IL-12, and IFN-γ		(Shirani et al., 2020)
MbovP503			Bind tight junctions, cross the epithelial barrier, and help in the colonization process		(Zhu et al., 2020b)
P26, Vsps, and VpmaX			Adhesion and pathogenesis of *M. bovis*		(Khan et al., 2017)
Methylenetetrahydrofolate-tRNA-(uracil-5-)-methyltransferase (TrmFO)			Cytadhesion to EBL cells		(Perez-Casal, 2020)
MBOV RS03440 (P27)			It is novel fibronectin (Fn)-binding, immunogenic adhesin of *M. bovis*		(Chen et al., 2018)
MbovNase			Essential for cytotoxicity, apoptosis, nuclease activity, and nuclear translocation		(Perez-Casal, 2020)
Variable surface protein of *Mmm* (Vmm)		*Mmm*	Enhances colonization and adaptation to the host tissue tropism at different stages of infection Plays a role in adhesion and immunomodulation		(Persson et al., 2002)
Glycerol- 3-phosphate oxidase (GlpO)			A major virulence factor due to its ability to release host cell-damaging H_2_O_2_ in the presence of glycerol and it can be used as vaccine candidate		(Pilo et al., 2005)
Lipoprotein (LppQ)			A highly antigenic lipoprotein specific to *Mmm*, and it is suitable as a diagnostic marker		(Anonymous, 2000)
(rP19 protein)			Interact with international standard serum against CBPP Adheres to EBL cells		(Zhou et al., 2016)
Serine protease S41		*Mmc*	Responsible for the caseinolytic activity Its inactivation causes obvious shifting in the expression or secretion of 17 predicted surface-exposed proteins		(Ganter et al., 2019)
MSCP136, MSCP636			Used to prepare a standardized cocktail ELISA protocol with a specificity and sensitivity of the novel cocktail ELISA were 96.4% and 85.6%, respectively		(Heller et al., 2016)
MSC_0894 (glycine hydroxymethyl transferase)			A cytoplasmic protein with an important role in the biosynthesis of purines, thymidylate, methionine, and other important biomolecules		(Krasteva et al., 2014)
MSC_0335 (ribosome binding factor)			Vital cytoplasmic protein for efficient processing of *Mycoplasma* 16S rRNA		(Krasteva et al., 2014)
PlsC (1-acyl-sn-glycerol-3- phosphate acyltransferase) GlpF-glycerol-3-phosphate oxidase			Involved in Glycerol metabolism pathway to release H_2_O_2_ and mediate cytoxicity		(Vilei and Frey, 2001; Pilo et al., 2005; Bischof et al., 2008; Bischof et al., 2009)
(MIB -MIP)			Prevent phagocytic uptake by convalescent IgG antibodies indicating a possible opsonization prevention mechanism		(Nottelet et al., 2021)
LppA (lipoprotein P72 of *MmmLC*) LppA (lipoprotein P67 of *Mmc*)		*MmmLC* *Mmc*	Two immunogenic lipoproteins showed a very high degree of similarity between these two mycoplasmas seems to fulfill the same structural functions		(Monnerat et al., 1999)
Immunoglobin binding protein (IbpM)		*M. pneumoniae*	A surface protein binds to different immunoglobulins (IgM, IgG, and IgA) produced by the host. It was demonstrated to produce cytotoxic effects in host cells in *M. pneumoniae* infection		(Blötz et al., 2020)
P1 adhesin			Form a complex with P30, P40, and P90 for performing different biological functions, such as gliding motility, receptor coordination, and binding a variety of host molecules such as Plg, Fn, vitronectin, and sialic acid		(Widjaja et al., 2020)
P116 adhesin			Similar to P1, but its exact function needs further investigations		(Chaudhry et al., 2007)
chaperones DanK and GroEL			Multifunctional and participate in the process of adhesion and dissemination		(Hagemann et al., 2017)
Mpn491			A secreted nuclease evades the NETs-mediated killing of neutrophils		(Yamamoto et al., 2017)
Mpn133			Essential for binding and nuclear localization of *Mycoplasma* proteins within the host cells		(Waites et al., 2017)
Alpha-enolase (Eno) Pyruvate kinase (PK) Glyceraldehyde-3-phosphate Dehydrogenase (GAPDH) Pyruvate dehydrogenases A-C Lactate dehydrogenase Phosphoglycerate mutase enzymes			Glycolytic enzymes play a potential role in *M. pneumoniae* adherence and invasion		(Gründel et al., 2016)
Cold agglutinins (cold reactive IgM autoantibodies)			Allow the pathogen to mask the pathogen-specific immune response. It is produced 1 to 2 weeks after infection due to the extensive sequence homology of the *M. pneumoniae* adhesin proteins and glycolipids of the cell membrane with mammalian tissues It can trigger autoimmune disorders that involve multiple organs and systems including, lung tissues, brain, kidney, myosin, liver, keratin, fibrinogen, and smooth muscle		(Waites and Talkington, 2004)
ADP-ribosylating and vacuolating cytotoxin			ADP-ribosylating activity provokes extensive vacuolization and critical cell death of mammalian cells Retains highly immunogenic epitopes		(Kannan and Baseman, 2006)
Community-acquired respiratory distress syndrome (CARDS) toxin			Induce macrophages to secrete TNF-α lead to pneumonic inflammatory cell infiltration Epithelial airway damage and plays a key role in *M. pneumoniae* brain infection through increase brain barrier permeability		(Li et al., 2019)
P97-like protein		*M. suis*	Involved in *M. suis* adhesion to RBCs		(Oehlerking et al., 2011)
Cold agglutinins; CAs)			Act mainly during the chronic stage of the disease Target the sialo-glycosylated regions of proteins on the RBC surface, and directly responsible for RBC agglutination, cyanosis, necrosis in the periphery of the blood circulation, and anemia		(Felder et al., 2010)
*Mycoplasma Suis* Gene product 1 (MSG1)/GAPDH			An erythrocyte adhesion protein interacts with Band 3 and glycophorin A in erythrocytes		(Song et al., 2018)
The variable lipoprotein (Vlp) family (VlpA, VlpB, VlpC, VlpD, VlpE, VlpF, and VlpG)		*M. hyorhinis*	Bind to both PK-15 and STEC cells. The binding increased in a dose-dependent manner and could be blocked by antisera against the rVlp proteins		(Xiong et al., 2016)
P37 protein			Facilitates metastases and invasiveness of various cancer cells *via* interaction with an epithelial cell adhesion molecule (EpCAM)		(Kim et al., 2019)
Green fluorescence proteins (GFP)			Essential marker to categorize the transformed cells and monitor transient gene transfer and expression in *M. hyorhinis*		(Ishag et al., 2016)
P110 (MgpC). P140 (MgPa and MgpB)		*M. genitalium*	antigen variation, which is correlated to optimization of adhesion, access to nutrients, survival in the host, and escape from the host defense mechanisms		(Wood et al., 2020)
Protein M			Extracellular, membrane-anchored prevent the antibody–antigen union, and could be part of an immunity evasion system based on antibody neutralization		(Hoelzle et al., 2014)
NADH-dependent flavin oxidoreductase (NFOR)		*M. hyo*	A metabolic enzyme related to oxidative stress A potential novel virulence factor, and so its contributing to *M. hyo* pathogenesis		(Xie et al., 2021)
P97 adhesin			A cilium adhesin that can undergo antigenic variation and is thus involved in evasion of the host immune response		(Minion et al., 2000)
P68 adhesin			A cilium adhesin that mediates the occurrence of inflammatory response and apoptosis		(Liu et al., 2019)
P216, P159, P146, P116, Mhp271, Mhp107 and Mhp683			ECM -binding adhesins		(Yiwen et al., 2021)
Variable adherence-associated (Vaa) antigen		*M. hominis*	The key adhesin of *M. hominis* mediates adherence of *M. hominis* to host cells		
P50t			Adheres to macrophages to evoke an immune response *via* the upregulation of TLR-2 expression and stimulate IL-23 production		(Goret et al., 2017)
*Mycoplasma* DnaK protein		*M. fermentans*	Binds with Poly-(ADP-ribose) Polymerase (PARP)-1 protein that plays a critical role in the pathways involved in recognition of DNA damage and repair Binds with (USP10), a key p53 regulator so reduces p53 anti-cancer functions.		(Benedetti et al., 2020)
Enolase (Eno)		*M.bovis* *M. hyo*	Acts as a multifunctional adhesin on the *M. hyo* cell surface for adherence to swine tracheal epithelial cells (STECs)		(Chen et al., 2019), (Perez-Casal, 2020)
Fructose-1,6-bisphosphate aldolase (FBA)			Binding, invasion and persistent infections		(Huang et al., 2019), (Perez-Casal, 2020)
Accessory proteins (HMW1, HMW2, HMW3, TopJ, MG218, and MG317 proteins)		*M. pneumoniae* *M. genitalium*	Cytadherence-related proteins help in terminal organelle maturation and clustering of other adhesion proteins to the tip structure, migration, and cell division		(Yiwen et al., 2021)
Organic hydroperoxide reductase (Ohr)			Novel proteins with hydroperoxidase activity on both inorganic and organic hydroperoxides
Heat shock protein (Hcp) P70		*M. pneumoniae* *M. hyo* *M. hominis*	Binding to host receptors		(Boulanger et al., 1995)
GAPDH		*M. hyorhinis M. bovis M. pulmonis* *M. genitalium M. hyo* *M. suis M. penetrans*	Important adhesin for promoting colonization Plasminogen receptor acting for extracellular matrix (ECM) degradation and promote systemic invasion Colonization and candidate for the future development of a potent vaccine against mycoplasmas infections		(Wang et al., 2021b) (Berry et al., 2017)
Recombinant α-enolase protein		*M. bovis* *M. suis*	Catalyzes the reaction of 2-phosphoglycerate to phosphoenol pyruvate Anti-α-enolase antibodies block adhesion		(Yiwen et al., 2021)
Methionine sulfoxide reductase (MsrA)		*M. genitalium, M. hyo* *U. parvum*	Antioxidant enzyme important for the maintenance of cytadherence		(Das et al., 2020)
Sialidase enzyme		*M. canis, M. cynos*, *M. molare*	Hydrolysis of sialic acid and participate in the destruction of ECM, colonization, and tissue invasion		(Robinson et al., 2017)
Elongation factor Tu (Ef-Tu)		*M. pneumoniae* *M. hyo*	On the surface of the human pathogen (*M. pneumoniae*), and the porcine pathogen (*M. hyo*) for Fn-binding adhesins and the ciliary border of the airway		(Widjaja et al., 2017)
Biofilm Formation		*M. pulmonis M. bovis*, *M. hyo M. agalactiae* *M. dispar, M. arginini*, *M. suis, M. alligatoris*, *MS*	Biofilm-forming mycoplasmas give them the ability to resist heat and dehydration which allow them to better survive in the environment		(Hoelzle et al., 2020)

### Mycoplasma Invasion Processes

Cell invasion is considered one of the most beneficial processes for mycoplasmas as it hides them away from the host immune system. Besides, living intracellular may enable them to pass through different body barriers such as the mucosal epithelium, get their nutritional requirements, and avoid the harmful effect of antibiotics (Vogl et al., 2008).

Mycoplasmas and ureaplasmas are the most frequently recognized intracellular pathogens in humans (Ferreira et al., 2021). *M. bovis* can invade different cell types such as T and B cells, monocytes, dendritic cells, NK cells, red cells, hepatocytes, cholangiocytes, renal tubular cells, facial nerve cells, etc. This invasion is beneficial to *Mycoplasma* in inducing inflammatory lesions, suppressing proliferation of immune cells, moving down from upper to lower respiratory tracts, and further spreading to other tissues from the lungs (van der Merwe et al., 2010) with the help of various invasive enzymes. Several previous reports have recorded the invasion and survival of various strains of *M. bovis* JF4278 and L22/93 (Bürgi et al., 2018) and Mb1 and Mb304 (Maina et al., 2019) in primary bovine alveolar macrophages (Suleman et al., 2016). In addition, *M. bovis* survival in necrotic lung lesions for long periods was reported, even in the presence of large numbers of neutrophils and macrophages. (Khodakaram-Tafti and Lopez, 2004). Other intracellular mycoplasmas also include *M. penetrans*, *M. pneumoniae, and M. genitalium* (Baseman et al., 1995), *M. suis* invasive strain (Groebel et al., 2009), and *MG* (Vogl et al., 2008).

Invasion of the mammary gland’s epithelium by *Mycoplasma* pathogens is a critical determinant for inducing mastitis and is associated with an altered immune response (Sordillo and Raphael, 2013). In this concern, these microbes can invade the gut lining epithelium following enteric infection that leads to their invasion into the host *via* the bloodstream and lymphatic (Nakagaki et al., 2018). More importantly, when the invasion is associated with immune depression of the host, bacterial dissemination to other organs takes place, including the mammary gland (Young et al., 2015). As such, live microbes can be detected in the bloodstream of animals (Zecconi et al., 2020) and humans (Aagaard et al., 2014; Whittle et al., 2018). An *in vitro* infection model showed the invasion of mammary gland epithelial cells has been established using 3 bovine epithelial cell lines (Josi et al., 2018).

To date, numerous mycoplasmas have been reported to produce invasion-related enzymes, such as proteases, nucleases, sialidases, antioxidant enzymes, and hyaluronidases. Nucleases, as important factors for mycoplasmas, are essential for degrading host nucleic acids; thereby having a critical role in growth, survival, persistence, and pathogenicity (Yiwen et al., 2021). For instance, Mpn491 secreted nuclease of *M. pneumoniae* (Yamamoto et al., 2017) and the major membrane nuclease (MnuA) of *M. bovis* (Mitiku et al., 2018) can degrade NETs and evade the killing ability of neutrophils. Proteases possess immunoglobulin (Ig) degradable capacities, as [*Mycoplasma* immunoglobulin binding (MIB) protein- *Mycoplasma* immunoglobulin protease (MIP)] (MIB-MIP) system to degrade IgG antibodies. *Mmc* carries the MIB-MIP system that exerts serine protease activity, followed by complete cleavage of IgG, thereby contributing to the evasion of the host immune system (Nottelet et al., 2021). Sialidase and neuraminidase are pathogenic enzymes for hydrolysis of sialic acid, destruction of extracellular matrix (ECM), tissue invasion, and apoptosis (Robinson et al., 2017). *MG* shows tropism to ciliated respiratory epithelium, then evades the mucociliary barrier followed by cell invasion (Matyushkina et al., 2016). It undertakes the invasion through penetration. Eventually, it resides intracellular, causing chronic or latent infection (Rüger et al., 2021).

Generally, after the invasion, mycoplasmas evolve and adapt to their parasitic intracellular life, they have slow intracellular growth rates compared to other extracellular bacteria. Their slow intracellular growth rate is mainly allowing them to hide and evade the host immune system (Rüger et al., 2021).

### Generation of Secondary Metabolites

Among the secondary metabolites, H_2_O_2_ is considered a critical virulent factor of *Mmm* (Wadher et al., 1990), but there is no direct correlation between the ability to produce H_2_O_2_ and virulence in *M. bovis* (Zhao et al., 2017) and *M. agalactiae* strains. In addition, hydrogen sulfide (H_2_S) is a novel potential virulence factor of *M. pneumoniae* (Shimizu, 2016); Nitrative stress markers are reported to be a potential virulence factor of both *Mmm* and *M. bovis* (Schott et al., 2014). A large amount of H_2_O_2_ can be produced by *M. dispar*, a biofilm-producing bovine respiratory pathogen with 23 identified potential virulence genes (Chen et al., 2019).

**Figure 3:**
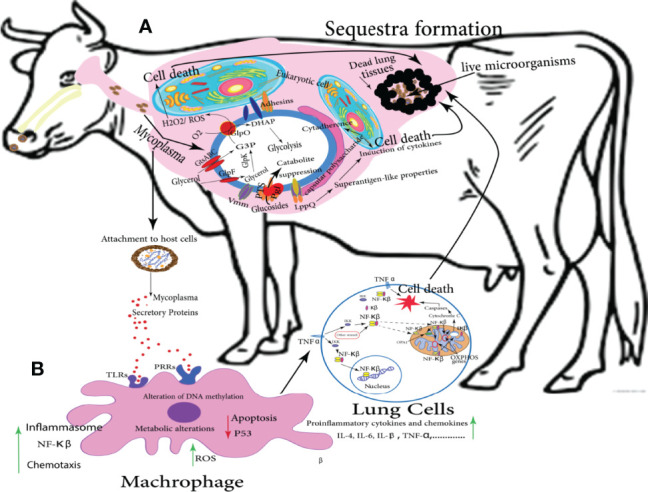
Potential metabolic pathways of *Mmm* involved in CBPP and sequestra formation: the following graph simulates the brief scenario that takes place *in vivo* and eventually leads to the occult problem of sequestra formation; **(A)** illustrates the interaction between mycoplasmas and lung eukaryotic cells following numerous pathways and release many metabolites that induce the targeted cell death. **(B)** showed the role of secretory proteins to stimulate the body immune cells to be directed toward the lung, activating different metabolic pathways including NF-κB, and stimulating a group of proinflammatory cytokines such as IL-4, IL-6, IL-β, TNF, etc. that is usually ended by cell death. Eventually, a collection of dead lung tissues containing live *Mycoplasma* pathogens called sequestra formation could be distributed in the lung (Di Teodoro et al., 2020).

In cattle, reactive oxygen species (ROS) is released by pulmonary phagocytes in the case of *Mmm*, and it damages the host cells. Numerous pathways have disturbed the integrity of cellular membranes, and/or indirectly enhanced the NF-κB pathway, thereby contributing to sequestra formation as shown in **Figure 3** (Karin and Delhase, 2000). Glycerol is consumed through Glycerol transporter system ATP-binding cassette (GtsABC) and the glycerol-3-phosphate oxidase (GlpO) (Pilo et al., 2007), both of them participate in glycolysis and production of ROS and H_2_O_2_. Eventually, it results in cytotoxicity and cell death. The polysaccharide capsule participates in *Mmm* persistence and induces cytokine production as shown in **Figure 3A** (Weldearegay, 2015).

The interaction of bovine lung cells with *Mmm* begins by attaching to mucous membranes of the respiratory epithelium followed by secretion of microbial secretory proteins that combine with specialized receptors on the surface of respiratory epithelial cells (Borchsenius et al., 2018).

Innate immune cells particularly macrophages, neutrophils, and natural killer cells are capable of recognizing pathogen-associated molecular patterns (PAMPs) of mycoplasma *via* toll-like receptors (Qin et al., 2019). Despite lacking a cell wall, mycoplasmas can interact with PAMPs (Demento et al., 2011). LAMPs such as MALP-2 (macrophage activating lipopeptide-2) and M161-Ag can induce TLR2 and TLR6 (Kumar et al., 2013). So far, LAMPs of *M. pneumoniae* can activate TLR1, TLR2, and partially TLR6 (Suzuki et al., 2003). In addition, other triacylated lipoproteins often stimulate TLR 1 and 2 but are TLR6 independent (Meylan et al., 2004). The subsequent inflammatory cascade begins with pathogen recognition by innate immune cells’ pathogen-associated molecular patterns (PAMPs) by interacting with specialized Pattern recognition receptors (PRRs) called Toll-like receptors (TLRs) (Demento et al., 2011). It also recognizes other emerged signals during tissue or cell damage that are usually known as danger-associated molecular patterns (DAMPs) (Medzhitov, 2007). This binding promotes the stimulation of macrophages which in turn induces the expression of pro-inflammatory cytokines and chemokines and the production of ROS. Additionally, the repression of p53-dependent apoptosis takes place. Eventually, it induces macrophages to produce TNF-α which acts for induction of the NF-κB inflammatory pathway (Borchsenius et al., 2020), a transcription factor that consists of a tri-subunit complex (P65, P50, and IκB) and exists in an inactive form in the cytoplasm. The activation of NF-κB only occurs when TNF-α attaches to TNF receptors. Then NF-κB activation inside mitochondria triggers cytochrome C release and the cell death occurred subsequently **Figure 3B** (Albensi, 2019).

### Antigen Variation

To date, only a few of the surface lipoproteins from *Mmm* have been studied thoroughly. LppA (p72), LppB, and LppC are highly conserved lipoproteins that are present in closely related species within the *M. mycoides* cluster (Sacchini et al., 2011). Vmm is a small surface protein shown to have a variable expression pattern (Persson et al., 2002). LppQ is a highly antigenic lipoprotein specific to *Mmm* (Perez-Casal et al., 2015). Thorough characterization studies and the development of a recombinant ELISA built upon LppQ antigen showed that it is a suitable diagnostic marker. *M. mycoides* cluster contains many candidate proteins such as the putative ATP-binding cassette (ABC) transporter and 187 predicted surface proteins of *Mmm*. More antigens than just LppQ, can trigger antibody-mediated immune responses, are useful in diagnostic applications. Combinations of such antigens could thereby offer a higher specificity and sensitivity than existing methods by adding discriminative power to the current LppQ based ELISA while circumventing cross-reactivity compared to whole-cell antigen-based methods (Krasteva et al., 2014).

PARCELs (Palindromic Amphipathic Repeat Coding ELements), are a set of widely distributed and repeated protein domains or genes that were probably gained and/or exchanged through HGT. They can be disseminated by multiple gene-centric vehicles (ORFs) carrying these elements for enhancing accessory gene pools, connecting genomes of various clades, and sharing common habitats (Röske et al., 2010). A tandem repeat pattern of 25 residues was initially reported in the LppQ lipoprotein presented on the surface of *Mmm*. Repeats of this category show considerable sequence variation among individual copies on the surface of the *Mycoplasma mycoides* cluster (Röske et al., 2010). LppC is an immunodominant antigen of *Mmm*, its amino acid sequence and its precursor showed similarity with two *Mmm* lipoproteins (LppB and LppQ). The N-terminal domain of the mature LppC seems to be surface exposed, but the C-terminal domain presented an integral membrane structure (Pilo et al., 2003).

Variable surface proteins (VSPs) are major highly immunogenic lipoproteins. The expression of these proteins can be switched on or off corresponding to gene reassortment induced by environmental change. Therefore, the surface antigenic phenotypes are modified to evade the host immune response. For example, in the genome of *M. bovis* type strain PG45 (American strain), the cluster of the vsp gene family has 13 genes (Clampitt, 2021), however, only 2 genes are expressed each time, the remaining genes remain silent. Furthermore, the size of proteins is kinetically regulated (Lysnyansky et al., 1999). In the genome of the Chinese *M. bovis* HB0801 strain, the vsp gene family has only 6 genes (Qi et al., 2012). Notably, the whole parasitic intracellular life of mycoplasmas is difficult to follow, because they differ from other bacteria for their unique small size and lack of a cell wall, and so their intracellular inhabitance as silent parasites has a substantial impact on cellular metabolism and physiology and immune evasion (Benedetti et al., 2020). More interestingly, their genetic evolutions have resulted in rapid modifications in their cellular membranes due to the previously mentioned considerable variations in VSPs. Also, the membrane lipid phase variations of distinct membrane surface proteins are crucial for adhesion and intracellular colonization. For instance, sequence variations and alterations in the structural domains encode surface cytoadherence proteins (Ferreira et al., 2021).

### Biofilm Formation

Biofilm formation by *Mycoplasma* species can increase *Mycoplasma* environmental persistence and survival. *M. bovis* has been confirmed to form a biofilm. Due to the high variation of VSPs, there is a big difference in the ability for biofilm formation among different *M. bovis* strains. The biofilm may induce resistance to dryness and heat indicating the enhancement of ability for environmental survival (Zbinden et al., 2015).

Mycoplasmas biofilm formation has been identified on both biotic and abiotic surfaces. Heterogeneously functional microcolonies are combined together to form one complex by bacterial polymeric substances such as polysaccharides, lipids, proteins, and extracellular DNA (Raymond et al., 2018). Mycoplasmas take the advantage of these biofilms as resistance to different environmental stressors such as antibiotics, antibodies, and host defense. Another advantage for *M. bovis* is to boost its environmental persistence, while inside the host leads to chronic infection (McAuliffe et al., 2006), whereas, exacerbating acute infection causes host and tissue damage after planktonic free cells are liberated from the biofilm causing host and tissue damage (Yiwen et al., 2021).

For *M. pneumoniae*, the more biofilms mature, the more cells encounter more morphologic changes. Additionally, H_2_O_2_, H_2_S, and CARDS toxin levels reach the peak at the early stage of biofilm formation but they will decrease over time indicating that the virulence of *M. pneumoniae* often reduces during the chronic infection stage (Feng et al., 2020). Microcolonies, the biofilm-forming unit, could also be identified *in vivo* in experimentally infected animals and were observed in *M. suis* by electron microscope on vascular endothelial cells (Sokoli et al., 2013). Raymond et al. also observed many ultrastructure molecules on the ciliated epithelium of the respiratory tracts in *M. hyo* infected pigs, they are essential for biofilm formation on abiotic surfaces (Raymond et al., 2018). For the first time, Awadh and coworkers have used scanning electron microscopy and confocal laser scanning microscopy to generate a 3-D image of *M. fermentans* biofilm architecture structure as a thin monolayer of cells to several layers thick which contain water channels allowing the diffusion of nutrients and oxygen (Awadh et al., 2021). When *Mmm* attaches to a solid surface, it can produce biofilms. Several extracellular binding adhesins such as pyruvate dehydrogenase were upregulated when it is included in adherent biofilm, and thus, the adherence process is essential and plays a key role in biofilm formation and commencing disease (McAuliffe et al., 2008).

### Role of Mycoplasmas Secretory Proteins

#### Pathogenesis and Immunity

The secretory proteins usually are toxins, adhesins, and virulence determining enzymes that participate in cellular adhesion, invasion, proliferation, and inhibition of host defense. Therefore, they play a critical role in bacterial infections. The secretome is the whole proteins secreted by bacterial cells. For *Mycoplasma* species, the secretome research just began. By using the secretome techniques, 27 secretory proteins have been preliminarily identified for *MS* (Rebollo Couto et al., 2012). *M. bovis* was first identified to have at least 60 secreted proteins (Zubair et al., 2020a). Later using an improved proteomic technique, 178 secreted proteins were identified and 79 differential secretory proteins were determined between *M.bovis* virulent HB0801 (P1) and attenuated HB0801-150 (P150) strains (Zhang et al., 2021a). However, there are only a few reports relating to secretory proteins of other *Mycoplasma* species.

*M. hominis* P80 is the first known secreted protein of *Mycoplasma* with a type I signal peptide sequence (Hopfe et al., 2004). For *Mmm*, glycerol phosphate oxidase was identified in the supernatant of *Mmm* culture and this enzyme can cause host cell damage and induce an immune response (Pilo et al., 2005). *Mycoplasma* nucleases were first reported by Razin et al. (1992). Remarkably, nuclease activity was membrane-associated, for instance, *M. pulmonis* has substantial DNase activity exposed on the cell surface (Minion and Goguen, 1986). One secretory nuclease encoded by *M. bovis* MBOV_RS02825 has been identified to degrade NETs (Zhang et al., 2016). Another secreted protein MbovP280 of *M. bovis* can induce apoptosis of macrophages through CRYAB (Zhao et al., 2021a). For *M. hyorhinis*, a 200 kDa secretory protein was confirmed to inhibit the cytotoxicity of T cells and mitotic activity induced by lipopolysaccharide (Teh et al., 1988).

As it is known, although mycoplasmas have some molecules associated with secretory systems such as SecA, SecY, SecD, DnaK, P36, lepA, and SecE in *M.hyo* (Leal Zimmer et al., 2020), SecA, SecD, SecE, SecG, SecY and YidC in *M. fermentans* (Rechnitzer et al., 2011), SecA, SecG, SecE, FtsY, LspA, SecD, ffh, secY, YidC in *Mcc* (Chen et al., 2017) and SecA, SecD/F, SecE, SecG, VirB4 (T4SS ORF from ICE), and YidC in *M.bovis*, they don’t have complete Sec or Tat systems (Qi et al., 2012). Regarding the secreted mechanisms, two models have been proposed. The first is a dual secretion model shown by the secretory proteins with a dual nature as both membrane and secretory proteins. These proteins are membrane proteins when the type I signal peptide sequences of the precursor proteins are inserted into the membrane, while they become secretory proteins after the signal peptides are cleaved (Hopfe et al., 2004). The location at the membrane may help *Mycoplasma* adapt to environmental change; while the secretion would make them contribute more flexibly to pathogenesis and immune response. For the non-typical secreted proteins without the type I signal peptide, the extracellular vesicles (EVs) release model is proposed. Six *Mycoplasma* species including *Mmm*, *Mmc*, *Mcc*, *M. agalactiae*, *M. fermentans*, and *M.bovis* have been demonstrated to produce EVs under nutritional stress and the proteins in EVs include major components involved in *Mycoplasma* -host interaction (Gaurivaud et al., 2018). The capsular polysaccharides of *M. pneumoniae* (Liu et al., 2012b) and *Mmm* (Pilo et al., 2007) have a potent cytopathic effect that can lead to host cell death (**Figure 3A**).

Exotoxins previously were considered absent in the Genus *Mycoplasma.* Now, community-acquired respiratory distress syndrome (CARDS) toxin is a membrane-associated, ADP-ribosylating, and vacuolating substance. It was identified by Kannan and Baseman (Kannan and Baseman, 2006). In *M. pneumoniae* pneumonic patients, a significant seroconversion has been identified, demonstrating that CARDS toxin can be synthesized *in vivo* with highly immunogenic power. But, when *M. pneumoniae* was cultured with host cells, the toxin production raised significantly compared to using an inanimate medium for *in vitro* culture. These findings assure that the toxin synthesis depends on the interaction between host cells and *M. pneumoniae* (Waites et al., 2017). On the other hand, a few mycoplasmas can secrete hemolysins, which cause erythrocytes lysis *via* pores formation on the cell membrane. For example, *U. parvum* and *U. urealyticum* display hlyA and hlyC, respectively (Marques et al., 2016).

#### Common Proteins in Secretomes of Different Mycoplasma Species

Orthologs are genes derived from a single ancestor gene in the compared species, while paralogs are genes related through duplication in the same species. In other terms, paralogs are homologous genes that appear in single genome analysis. Since orthologs have equivalent functions, the comparative study of mycoplasmas secretomes is expected to produce a breakthrough in the exploration of common secreted proteins as novel biomarkers for mycoplasmas’ diagnosis and vaccine development (Zhang et al., 2021a). For example, *M. bovis* and *Mmm* are not only the most pathogenic cattle mycoplasmas, but also responsible for significant economic losses (Nicholas et al., 2008). They cause respiratory diseases with similar clinical and pathological symptoms. From the genome level, it was found that horizontal gene transfer (HGT) between them might occur (Citti et al., 2018). It would be probable for these events to happen when both pathogens infect the same hosts (Dudek et al., 2021). The comparative study of *M. bovis* and *Mmm* secretomes would reveal the homologous and unique secretory proteins and help understand the *Mycoplasma* evolution in cattle and develop common diagnostic reagents and vaccines for cattle.

The virulence-related factors identified at the protein level so far have been summarized in **Table 1**.

#### Evolution of Phylogenetically Related Mycoplasmas

**Figure 4:**
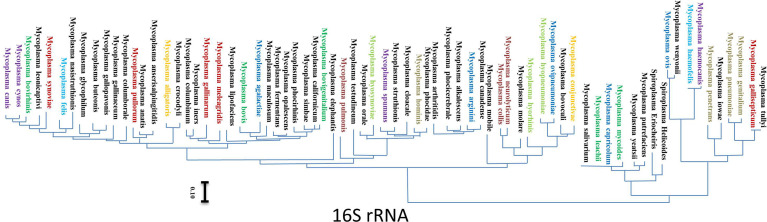
Evolutionary phylogenetic tree generated from 16S rRNA sequences: bars indicate distances under the corresponding tree. The pathogenic mycoplasmas of the major importance were colored and gathered in groups according to their main host, each group has a different color. The tree branches show the distance between neighboring mycoplasmas species (Chen et al., 2022).

A more complete view of mycoplasma evolution came from the comparative analysis of 16S rRNA oligonucleotide catalogs (Woese et al., 1980). Based on a 16S rRNA (**Figure 4**) sequence comparison, *M. hyo* and *M. flocculare* are known to be closely related. They are similar to the situation found in the genomes of the two closely related species *M. pneumoniae* and *M. genitalium*, whose genomes can be divided into segments with highly conserved gene organization, although the segments are arranged differently (Stemke et al., 1992), (Himmelreich et al., 1997). To identify the unique and common genes of *M. flocculare*, *M. hyo*, and *M. hyorhinis* and to explain the different behaviors of these species in swine respiratory tracts, Siqueira et al. took advantage of “the bidirectional best hit (BBH) approach”. Their results suggest that *M. flocculare* and *M. hyo* or *M. flocculare* and *M. hyorhinis*, share several ORF clusters (OCs) genome pairs, which can partially be attributed to HGT (Siqueira et al., 2013). Phylogenetic studies using sequence analysis of 16S rRNA genes resulted in 99.9% similarity between *Mmm* and *Mmc*, because of that they were included within a single mycoplasmas subspecies (*M. mycoides subsp. capri*) (Pettersson et al., 1996).

### The role of Phages and Prophages in Sustaining the Virulence in Mycoplasmas

Bacteriophages/bacteria eaters or “phages” are viruses that infect bacteria. Most of them kill their bacterial hosts. Phages have been identified for four mycoplasmas, including Mycoplasma arthritidis (phage MAV1), Mycoplasma hyorhinis (phage Hr1), Mycoplasma pulmonis (phage P1), and Mycoplasma bovirhinis (phage Br1) (Voelker and Dybvig, 1998). Of which, MAV1 was the only known virulence-associated phage. It is found in certain highly virulent strains of Mycoplasma arthritidis. In parallel, the polymorphisms within MAV1 prophage integration sites and within the prophages themselves may help to identify phylogenetic relationships among virulent *M. arthritidis* strains (Washburn et al., 2004).

The phage genome (prophage) is usually incorporated into the bacterial genome and transmitted vertically during replication. Prophages play a vital role in bacterial evolution, virulence determination, population shaping, and genetic transfer *via* horizontal gene transfer (HGT), which in turn influences bacterial traits. For example, *Mycoplasma* species have been proved to maintain a large complex prophage-like genomic island for the first time that carries a highly conserved gene cluster. This gene cluster is expressed in mycoplasma cells retaining resistance to three antibiotics (aminoglycosides, kanamycin, and neomycin) (Lysnyansky and Borovok, 2021).

The airway epithelium is the main place where exotic and commensal microbes interact between themselves and the host. Notably, the pulmonary surfaces (nasal and bronchial epithelium) are covered by mucus which contains mucin glycoproteins and nutrients. This environment is favorable for commensal bacteria and phage symbionts; therefore, these phages are critical for regulating bacterial populations in almost every niche (Tzani-Tzanopoulou et al., 2021). As such, it is well-known that phage communities are ampler in mucosal surfaces compared to other non-mucosal parts, and so these phages ensure a sustainable source of virulence evolution for different Mycoplasma species (Meyer, 2013)

In particular, *M. hyosynoviae* NPL4 strain is one of the most heavily phage-infected strains of *Mycoplasma*. Its genome was reported to contain two complete as well as one incomplete prophage sequences. Nevertheless, *M. hyosynoviae* can defend against invasion by phage as two of its strains contain a CRISPR-Cas system that keeps them resistant to infection. Interestingly, several prophage genes were discovered present within the genomes of *M. hyosynoviae* with significant similarity to its related species, *M. arthritidis* MAV1 phage (Bumgardner et al., 2015).

On the other hand, Bacteriophage-mediated immunoscreening is a promising field using an appropriate vector system. It offers a rapid and simple technique for the identification and immediate testing of putative candidate vaccines. For example, *Mmm* DNA vaccine, in which a whole-genome library was cloned into a bacteriophage λ ZAP, and then the phage library was plated on Escherichia coli cells (March et al., 2006).

## Horizontal Gene Transfer (HGT) and Transmission of Genetic Information

Genome is dynamic in nature, and therefore epigenetic modifications have a great impact on it. To a large extent, genes may be lost, and/or the non-coding genomic regions may expand or shrink over a period of time. Furthermore, selective pressures over different genomic positions make them evolve differently. Also, epigenetic alterations in cancer include changes in DNA methylation that influence gene expression as mycoplasmas predispose their host to carcinogenesis (previously mentioned in the human infection section). Eventually, genes can be obtained *via* duplication within the same genome or acquisition from another organism through HGT which is considered the main regulator of microbial diversity. It is almost the final result of the infectious spread of mobile genetic elements (MGEs) in bacteria such as integrative and conjugative elements (ICEs), bacteriophages, and plasmids (Hall et al., 2017). In this concern, prevailing reports suggest that the transfer of mobile genetic elements (MGE) may represent only the tip of the iceberg (Blesa et al., 2017).

Remarkably, large chromosomal fragments can be passed across genomes. Besides, their subsequent consecutive reconstitution will be more effective, prominent, and complex than first imagined *via* unknown mechanisms (Husain et al., 2017). In 2014, the conjugal transfer of large chromosomal regions among ruminant *Mycoplasma* species has been demonstrated for the first time which had never been recognized in mycoplasmas’ research field. Intriguingly, it clearly illustrated the inter-species transmission of these pathogens between different hosts (Dordet-Frisoni et al., 2014). *Mycoplasma* chromosomal transfer (MCT) is a newly documented strategy that induces enormous exchanges of genomic materials. This potent mechanism has a profound impact on genetic rearrangement that reshuffled parental characteristics and created mosaics. It depends on the functional integrative conjugative element (ICE) in one partner that plays a part in the horizontal acquirement of small or large chromosomal segments as in the case of *M. bovis* (García-Galán et al., 2021). This has been most clearly demonstrated for *M. agalactiae*, numerous chromosomal DNA fragments and generated offspring comprised of a variety of genomic assortment, each proven to be unique. These gave us up to 17% of the exchanged genome. The genome has been predicted using comparative genomics that almost 18% of its genetic material has undergone HGT with mycoplasmas of the unrelated *M. mycoides* cluster (Dordet-Frisoni et al., 2019). A large number of ICEs copies were detected in several sequenced *Mycoplasma* genomes (Tardy et al., 2015), raising the likelihood that these simplest bacterial pathogens may be capable of conjugation mainly during inter-species transmission of pathogenic mycoplasmas (**Table 2**) (Dordet-Frisoni et al., 2019). Faucher et al. (2019) have established an advanced model to evaluate antimicrobial resistance of enrofloxacin in mycoplasmas after that; they performed a “genome-scale analysis” of major and minor determinants that lead to antimicrobial resistance. A novel protocol, for optimized conjugation in the case of *M. agalactiae* and *M. bovis*, has been adapted. It allows the horizontal transfer of ICE or chromosomal fragments carrying antibiotic resistance genes with estimating the frequency of conjugations. It can be modified also for the other *Mycoplasma* species (Sagné et al., 2021). Citti and Blanchard stated that the collected experimental data support their preliminary *in silico* predictions, and also verified that MCT has been shaping various *Mycoplasma* species with a mosaic-like genome (Citti and Blanchard, 2013), these mycoplasmas *spp.* might possess the ability to join together and support HGT strategy (**Table 3**).

**Table 2:** Inter-species transmission of *Mycoplasmas and Ureaplasmas*.

Host	*Mycoplasma* pathogens	Reference
Human	*M. pneumoniae*	(Chalker et al., 2012)
	*U. urealyticum*, *M. genitalium*, *M. hominis*, *M. fermentans*, *M. penetrans*	(Benedetti et al., 2020)
	*M. arginini*, and *M. arthritidis*	(Zella et al., 2018)
	*M. pirum*	(Wohlman et al., 2001)
	*Ureaplasma species* (*U. urealyticum and U. parvum*)	(Glaser and Speer, 2015; Robertson et al., 2002)
	*M. suis*, *M. haemofelis*, and *M. ovis*	(Maggi et al., 2013b)
	*M. bovigenitalium* and *M. agalactiae subsp. bovis*	(Tourtellotte and Lein, 1976)
	HM (aka *hemoplasmas*)	(Díaz-Sánchez et al., 2019)
Bovines	*Mmm*	(Jores et al., 2020)
	*M. bovis*	(Caswell et al., 2010)
	*M. dispar* and *Ureaplasma diversum*	(Howard, 1983)
	HM (aka hemoplasmas)	(Díaz-Sánchez et al., 2019)
Ovines	*M. ovipneumoniae*	(Besser et al., 2019)
	*M. ovipneumoniae* and *M. arginine* combined infection	(Niang et al., 1998)
	*M. bovigenitalium* and *M. agalactiae subsp. bovis*	(Tourtellotte and Lein, 1976)
	*M. ovis* and ‘Candidatus *M. haemovis*’	(Machado and André, 2019)
Caprines	*Mcc*	(MacOwan and Minette, 1976)
	*M. ovipneumoniae*	(Jaÿ et al., 2020)
	*M. agalactiae*	(Santos et al., 2015)
Equines	*M. felis*	(Kinoshita et al., 2020)
	Unidentified mycoplasmas, (strains N3 and NI1) that cross-reacted with strains of *Mmm and Mmc*	(Lemcke et al., 1981)
	HM species (Candidatus *M. haemobos-* like species)	(Dieckmann et al., 2010)
	Unidentified *Mycoplasma* species	(Manguin et al., 2020)
Swine	*M. hyo*, *M. flocculare*, and *M. hyorhinis*	(Siqueira et al., 2016)
	*M. suis*, *U. parvum*, and *M. haemosuis*	(Fu et al., 2017)
Pet animals (dogs and cats)	Around 15 different *Mycoplasma* species have been isolated as commensal	(Chalker, 2005)
Canines	*M. canis*, *M. cynos*, *M. edwardii*, and *M. spumans*	(Jambhekar et al., 2019)
	*M. hemocanis* (Mhc) and Candidatus *M. haematoparvum* (CMhp)	(Sharifiyazdi et al., 2014)
Felines	*M. haemofelis*	(Tasker et al., 2003)
	Candidatus *M. haemominutum*	(Foley and Pedersen, 2001)
	Candidatus *M. turicensis*	(Messick et al., 1998)
	*M. felis*	(Foster et al., 2004)
	Candidatus M*. haematoparvum-like (CMhp)*	(Barker and Tasker, 2013)
Lab animals	*M. pulmonis*	(Booth et al., 2014)
	*M. neurolyticum*	(Findlay et al., 1938)
	*M. collis*	(HILL, 1983)
	*M. muris*	(McGarrity et al., 1983)
	*M. arthritidis*	(Constantopoulos and McGarrity, 1987)
Wild animals	*Mcc*	(Baziki et al., 2020)
	*M. ovipneumoniae*	(Jaÿ et al., 2020)
	*M. ovis*	(Boes et al., 2012)

**Table 3:** Non-specific host infection allows gene transfer between *Mycoplasma* species.

Mycoplasma species	Non-specific hosts	Reference
*M. ovis*	Human	(Maggi et al., 2013b)
	Rangifer tarandus species (Reindeer) (USA)	(Stoffregen et al., 2006)
	Mazama gouazoubira (gray brocket deer)	(André et al., 2020)
	Free-ranging Cervus nippon species (Spotted, Japanese deer) (Japan)	(Watanabe et al., 2010)
	Free-ranging B. dichotomus and O. bezoarticus deer species (Brazil)	(Grazziotin et al., 2011)
	white-tailed deer	(Boes et al., 2012)
*M. ovis*-like species	German horses	(Dieckmann et al., 2012)
	Iranian horses	(Kalantari et al., 2020)
*M. felis* strain Myco-2	Horse (Japan)	(Kinoshita et al., 2020)
*M. alligatoris*	alligators, caimans, and crocodiles (USA)	(Brown et al., 2001)
*M. testudinis* *M. agassizii*	Desert and spur-thighed tortoise (USA)	(Origgi and Jacobson, 2000)

Currently, genome sequences in databases are established for more than 60% of the known *Mycoplasma* species (>150) and for over 280 strains, in which almost half are available as a single circular chromosome. These figures show a fast increment, however, the already provided data guarantee a valuable source for mining total mycoplasmas genomes. Comparative genome analyses integrated with saturation transposon mutagenesis have been already established for over 20 years. The number of functional genes is likely to be closer to 450 based on synthetic genome studies. The remaining genes’ compartment was predicted to encode hypothetical proteins with little tendency to virulence factors (Citti et al., 2020). Phylogenetic analysis for the genome of many *Mycoplasma* species is distinct but sharing the same reservoir has contributed to the exchange of large DNA segments (Liu et al., 2012a). Bioinformatics analysis tools also predict HGT of the (MIB–MIP) system (previously mentioned in the *Mycoplasma* invasion processes section) between mycoplasmas infecting the same hosts, assuming that MIB–MIP is a shared system engaged in a worldwide strategy to elude the host immune system. Notably, MIP/MIB system was originally identified in *Mmc* (Nottelet et al., 2021). The identification of this system opens a new research route for a better understanding of the strategies exploited by minimal bacteria to escape the sophisticated immune systems of mammalian hosts (**Table 2**) (Arfi et al., 2016).

For the first time, an innovative technology of artificial genome was created as a minimal genome generated as a functionally competent artificial cell has been assembled by introducing a synthesized genome inside a cell envelope of a *Mycoplasma* designed with the help of transformation techniques (Cordova et al., 2016). Nowadays, genome transplantations have only been accomplished in atypical bacterial agents (Mollicutes). This modern technology will allow us to study comparative genomes of different mycoplasmas *spp.* which in role assist us in more accurate proteomics profiling and determining the potential essential common proteins (Baby et al., 2018).

## Immunity, Diagnosis, and Therapy

### Immune Response

The hallmark of *Mycoplasma* respiratory infection is the persistence of lung inflammation involving both innate and adaptive immunity. Recently, IL-17 has gained a lot of attention in respiratory *Mycoplasma* infection; it also has a hand in pathologic outcomes of lung infection (Luo et al., 2021b). Many recent studies, including a study, carried out in our lab, have proved that a variety of cells, particularly Th17 cells, in the lung can secrete IL-17. It contributes to respiratory *Mycoplasma* infection, as shown in our previous lab work using two groups of calves infected with the virulent HB0801 (P1) and attenuated HB0801 P150 strains of *M.* bovis (Chao et al., 2019). Peripheral blood mononuclear cells (PBMCs) also play a pivotal role to enhance innate immune response; Chao et al. have studied their transcriptome profiles. They found out 7 and 10 core differentially expressed genes (DEGs) in P1 and P150 groups, respectively. (Chao et al., 2019). Overall, the studies about immune response and pathogenesis concentrated on membrane proteins that also can be used as novel vaccine candidates (Krasteva et al., 2014).

Innate immunity plays a key role to control *M. bovis* infection, however, the pathogen has developed mechanisms to overrun and modulate apoptosis of bovine PBMCs and, thus, Gondaira et al. (2020) have evaluated the bovine mammary gland response following infusion of *M. bovis.* Somatic cells and bacterial cells counts in milk samples were increased; however, the proliferation of PBMCs and lymph node mononuclear cells (MNCs) of *M. bovis*-stimulated mammary glands was the same as unstimulated cells. Transcriptome analysis revealed that the mRNA levels of innate immune system-related genes in blood PBMCs, complement factor D (CFD), and tumor necrosis factor superfamily member 13 (TNFSF13) decreased. The mRNA levels of immune exhaustion-related genes, programmed cell death 1 (PD-1), programmed cell death-ligand 1 (PD-L1), lymphocyte activation gene 3 (LAG3), and cytotoxic T-lymphocyte- associated protein 4 (CTLA4)) of the milk MNCs in the infected quarter were increased as an indication of general immune suppression. While the mRNA levels of innate immune response-related genes of MNCs in the infected quarters were decreased (Gondaira et al., 2020). Much more deeply, the transcription of innate immunity-related genes in PBMCs (IL-17, IFN-γ, IL-27, and IL-36A) has been raised during *M. bovis* infection. These induce the triggering of T-cell subsets and cellular immune responses (Gondaira et al., 2021).

The immune response of mycoplasmas dictates what happens to them inside their niches. The primary *Mycoplasma* species that possess a prominent detrimental role worldwide is *M. bovis*, a major contagious pathogen in dairy and feedlot cattle that can suppress the host immune response during infection and develop a chronic inflammatory response that causes pathological immune damage in the target organs (van der Merwe et al., 2010). *M. bovis* develops several strategies to escape immune system elimination through inhibiting neutrophils, secreting a unique immunosuppressive peptide that inhibits the proliferation of bovine lymphocytes, and stimulating monocytes to produce anti-inflammatory factors. These factors cause apoptosis, suppress proliferation, and induce invasion of PBMCs leading to the persistence of chronic infection (Askar et al., 2021).

During *M. bovis* infection the host response itself contributes to the disease pathogenesis. It possesses superior strategies to elude host responses. Stimulation of both proinflammatory and anti-inflammatory cytokines takes place at the same time with skewed T-cell response accompanied by T-cell exhaustion in chronic infection with escaping immune clearance (Maunsell and Chase, 2019). Immunoglobulin-binding proteins are commonly known in many *Mycoplasma* species. They act to help the bacterial evasion of the host immune response (Arfi et al., 2016). To name a few, MBOVPG45_0375 (r0375) can bind to IgG and cause antibody neutralization to inhibit the antigen-antibody immune complex formation. MBOVPG45_0376, another membrane protein of *M. bovis* PG45 strain, is a novel IgG-cleaving protein that has a great impact on the interaction between *M. bovis* and host cells (Zhang et al., 2021c). P48, as an important virulence-related membrane protein of *M. bovis* involved in the adhesion process, its effect on EBL cells has been explored to further explain *M. bovis* infection mechanism. Remarkably, exogenous P48 protein inhibited EBL cells growth and induced similar apoptosis patterns as *M. bovis* infection, extracellularly and intracellularly (Wu et al., 2021).

### Vaccination

Betlach et al. (2021) have investigated the potential impact of multiple vaccinations on reducing *M. hyo* transmission and infection; they found that the three-dose of commercial bacterin vaccination strategy significantly reduced bacterial load in inoculated gilts and decreased *M. hyo* lung lesions at 28 dpi in challenged gilts, as well. Another recent study compared *M. hyo* response to infection by route of exposure, concluding that intratracheal exposure produced the highest percentage of *M. hyo* DNA-positive pigs and higher serum antibody response which can be considered during setting a vaccination strategy (Silva et al., 2021). Another study, comparing the convenience and economic benefits of vaccinating piglets with *M. hyo* at 3, 7, and 14 days of age, has found that *M. hyo* vaccination at 3 days of age has supreme advantages over 7 or 14 days of age (Vangroenweghe, 2021). Recently, many studies concerning *M. hyo* vaccines evaluation have estimated the efficacy of new bivalent and trivalent vaccines containing *M. hyo* confirming that this vaccine provided good protection against *M. hyo* challenge (Yang et al., 2021a; Yang et al., 2021b).

Innate immunity regulatory factors, mainly Mannose-binding lectins (MBL) for developing new vaccines, play a key role to resist foreign pathogens invasion including *Mycoplasma* through selective recognizing lectins on the surface of bacteria. To date, it constitutes the first line of innate immunity against infection through activating complement, phagocytosis, and opsonization (Zhu et al., 2021a). *MS* bacterin was used after adding different adjuvants that can induce innate immunity. Chitosan adjuvant has enhanced lymphocyte responses and interleukins upregulation with systemic protection after subcutaneous injection (Gong et al., 2020). *M. genitalium* is the causative agent of several sexually transmitted infections in animals and humans. Subtractive genomics and reverse vaccinology have been applied *in silico* identifying potential vaccine and drug targets against five strains of *M. genitalium*, 14 novel vaccine candidates and 2 novel drug targets were finally predicted (Nogueira et al., 2021).

In humans, *M. pneumoniae*-derived lipids and membrane lipoproteins play a critical role in the inflammatory responses. Using an antibody-neutralizing assay, it was demonstrated that TLR-4 is essential for *M. pneumoniae* lipid-induced TNF-α and IL-1β production. NF-κB-dependent pathways also are critical for pro-inflammatory cytokines secretion (Luo et al., 2021a). Numerous types of *M. pneumoniae* vaccines have been designed in the form of whole-cell vaccines (inactivated or live-attenuated), subunit vaccines (involving P1, P30, P116 proteins, and CARDS toxin), and DNA vaccines (Jiang et al., 2021).

CBPP is the major threat to the cattle industry in Africa, affecting almost 25 countries. Several novel experimental vaccines have been developed over the last 2 decades to improve the T1/44 live vaccine protection ability, but mostly they have aggravated the disease (Dudek et al., 2021). The subunit vaccines formulated with a combination of recombinant proteins of *Mmm* showed protection against challenge with the most virulent *Mmm* strain (Afadé) (Nkando et al., 2016). Meanwhile, *M. bovis* has spread now to most cattle-raising countries. Vaccination is the basic focus for infection control because of its increasing resistance to antimicrobial therapy, but commercial effective vaccines are currently absent. In our previous work concerning *M. bovis*, we concluded that the protection rate of the P150 *M. bovis* attenuated strain was 87.7%, and thus, it is a promising candidate for a live vaccine against *M. bovis* infection in cattle (Zhang et al., 2014). In our previous study, we discovered 10 core DEGs in the P150 *M. bovis* HB0801 attenuated strain, These DEGs can be used in further studies for improving attenuated P150 strain (Chao et al., 2019). Finally, our team has determined 79 differential *M. bovis* secretory proteins between the virulent P1strain and attenuated P150 strain (Zhang et al., 2021a).

### Nanotechnology for Diagnosis of Mycoplasmas Infection

The limitations of available options for *Mycoplasma* diagnosis highlighted a critical need for a new detection platform with high sensitivity and specificity. To achieve better detection efficiency, single-walled carbon nanotubes (SWCNT) coupled with colloidal gold-monoclonal antibody immunochromatographic strips (CGIC) have been used (Song et al., 2017).

As growing advanced fields, the loop-mediated isothermal amplification (LAMP) (Wang et al., 2019a) and multiple cross displacement amplification (MCDA) techniques combined with nanoparticle-based lateral flow biosensor (LFB) assay have been developed and evaluated (Wang et al., 2019b). These techniques are simple, reliable, and smart enough for the identification of *M. pneumoniae.* The LAMP-LFB assay specifically identified DNA templates of *M. pneumoniae*, and cross-reactivity with other pathogens did not occur (Wang et al., 2016; Yuan et al., 2018).

Previously, Biosensors have been designed using silver nanorod arrays (NA) for identifying *M. pneumoniae* in culture and throat swab samples. More interestingly, these biosensors are characterized by high specificity (95%–100%) and good sensitivity (94–100%) (Hennigan et al., 2010). Thereafter, Henderson et al. have established nanorod array-surface enhanced Raman spectroscopy (NA-SERS). It detects *M. pneumoniae* in true and simulated throat swabs at the qualitative endpoint of < 1 cell/μl with a sensitivity exceeding that of qPCR (Henderson et al., 2014) and high specificity and strain-typing capacity (Henderson et al., 2015). In addition, gold nanoparticles have been used for the rapid detection of *M. suis* in porcine plasma (Bai et al., 2018).

Today, the surface-enhanced Raman scattering (SERS) biosensor, a kind of ‘whole-organism fingerprint’, has been invented with a smart capability of identifying three *Mycoplasma* species *M. hominis, M. genitalium*, and *Ureaplasma urealyticum* (Berus et al., 2021). The most recent today’s technology in the field of *Mycoplasma* diagnosis using *M. pneumoniae* as a target pathogen has gained a highly sensitive DNA detection limit of 3.12 pg/μL. After 10 cycles of the PCR-coupled SERS method, it has shown enhanced detection ability. This technology is composed of a low-cost paper-based SERS substrate in the form of silver-nanowires (AgNWs) then coupled with PCR for rapid and sensitive *Mycoplasma* DNA determination (Lee et al., 2021).

### Nanotechnology for Mycoplasmas Therapy

Generally, mycoplasmas are susceptible to antibiotics that affect proteins including tetracycline, macrolides, Lincosamides, and phenicols or nucleic acid synthesis like fluoroquinolones. However antibiotic resistance sometimes develops against these antibiotics causing a decrease in the effectiveness of certain antimicrobial agents (McDermott et al., 2016). To overcome the development of multidrug resistance (MDR) of major pathogens that threaten humans, nanotechnology-based drug delivery systems have been emerging as a talented approach. They have been used to avoid the drawbacks of traditional drugs, decrease antimicrobial resistance, and open the hypothesis for new drug formulations (Algharib et al., 2020a). Nanoparticles (NPs) are common encapsulation materials with the great advantage of increasing intracellular accumulation of the drug to overcome bacterial resistance. For example, metallic and carbon nanotubes can be used for inhibiting bacterial biofilm formation as well as solid lipid nanoparticles as nanocarriers for antimicrobial agents that cannot be administrated as free drugs (Arana et al., 2021). In our previous work to combat the growing MDR, we have designed and optimized, for the first time, chitosan nanogel to encapsulate a rifaximin. It increases bioadhesion of rifaximin, and thus targeted release in the intracellular and extracellular bacterial infection sites (Algharib et al., 2020b) as in the case of *Mycoplasma* infection.

Basically, the nano-drug delivery system has many advantages not achievable by conventional drugs. The drugs can be encapsulated into nanoparticles and thereby increase their solubility, enhance their absorption and uptake by cells, target them to specific organs, and release them in a controlled manner as a response to specific stimuli (Andrade et al., 2011). Additionally, some nanoparticles have great potential in medical microbiology due to their antibacterial effects with low toxicity against the hosts (Djurišić et al., 2015). Several types of nanoparticles have been designed like rifampicin/poly (lactic-co-glycolic acid) nanoparticles for delivery to the lungs by nebulization, they need further research to be tested regarding their effectiveness against mycoplasmas infection (Andrade et al., 2013). At present, silver nanoparticles have received more attention in scientific research. Yang et al., (Yang et al., 2019b) have designed and evaluated, for the first time, the anti-*Mycoplasma* pneumonia potential of biosynthesized herbal-based (Zingiber zerumbet) silver nanoparticles in experimental mice. The lincospectin- zinc oxide nanoparticles (ZnO-NPs) are effective against *M. bovis* through destructive oxidative stress to bacterial cells and disrupt their metabolic activity thereby inhibiting their growth. Nowadays, Zn is available as a food additive since it can improve the immune system, prevent biofilm formation, and has low toxicity to human cells (Fathi et al., 2019).

TLR2 agonists, effective prophylactic antigens or immunotherapeutic agents for many pathogens, are located on the cell membrane surface to recognize microbial LPS or lipopeptides. These ligands can be used as molecular adjuvants with vaccines for providing a threat signal to keep a long-lasting adaptive immune response. This idea was represented by Franzoni et al. (2021) who used a lipopeptide based on *M. agalactiae* surface protein (Mag-Pam2Cys) that activated antimicrobial innate immunity by polarizing porcine macrophages. More creatively, these molecules can be loaded on a compatible nanocarrier to improve their invasiveness and therapeutic power.

## Discovery of Enigmatic Features of Mycoplasmas

In order to clarify the mystery regarding *Mycoplasma* virulence and its immune escaping ability, contemporary trends are directed toward genome transplantation and genome assembly focusing on the advancement of cutting-edge technologies (Labroussaa et al., 2019). Through using the most current technological devices, an important study on *M. genitalium* with a 580 kb genome, the smallest complete genome identified until now, has been conducted. They found that (55-73) % of the protein-coding genes are essential, and so they are considered the minimal set of genes essential for maintaining bacterial life (Hutchison et al., 1999). Another study on *M. genitalium* has identified 382 essential genes of the 482 protein-coding genes (Glass et al., 2006). The first synthetic *Mycoplasma* genome was created for *M. genitalium* with 583 kb. This technical achievement was a great step in the field of synthetic biology. More interestingly, the project of genome transplantation in bacteria has been established by changing one species into another (Lartigue et al., 2007). Afterward, a fully synthetic *M. mycoides* genome was transplanted into *M. capricolum* (Gibson et al., 2010), this genome was reduced to include the only essential genes for its life (473 genes) (Hutchison et al., 2016). These achievements will allow us to set and redesign future genomes for medical products by using computational tools (Rees-Garbutt et al., 2020). *M. pneumoniae* is another important bacterial model with a natural tropism to the human respiratory tract (Wodke et al., 2015). Indeed, cutting-edge technology is used to study *M. pneumoniae* proteome interactions with host cells using mass spectrometry which provides an accurate perception of the structural information on *M. pneumoniae* proteins (Kühner et al., 2009). Consequently, experimental validation of the whole metabolic network map has been conducted (Yus et al., 2009).

Other new promising directions were used to unravel the mycoplasmas panoply. To name a few, X-ray crystallography and cryo-electron microscopy tomography can explore the tridimensional structure of the various illusive virulence determinants for cytoadhesins of *M. genitalium and M. pneumoniae* (Vizarraga et al., 2021). It will be important to first confirm the cellular localization of these proteins using available fluorescent molecules e.g., mNeon71, or tags using electron microscopy. After that, a list of surface-exposed virulence candidates will be available that can be verified in *ex vivo* and *in vivo* biological systems (Jores et al., 2020). Also, MIB and MIP are surface proteins which present in the majority of *Mycoplasma* species. Cryo-electron microscopy showed how these proteins perform a “hug of death” strategy when bound to antibodies disrupting the antigen-binding sites (Nottelet et al., 2021). Acting to protect mycoplasmas from antibody-mediated agglutination, the MIB-MIP system is considered a landmark of mycoplasmas immune evasion.

Up to now, Micro RNAs (miRNAs) are key molecules that regulate gene expression *in vivo* with specific pathways. For instance, miR-509-5p negatively regulates the NF-κB pathway, thereby affecting the inflammatory response of *M. pneumoniae* in sheep (Zhu et al., 2021b). Additionally, the impact of protists/bacteria relationships has been rarely taken into account by microbiologists. Today, the understanding of protist evolution is highly dependent on prokaryotes, for example, HGT from *M. hominis* to symbiotic hosts allows adaptation of both species to new eukaryotes habitats (Henriquez et al., 2021). The ability of *M. hominis* and *Trichomonas vaginalis* (the most common sexually-transmitted protozoan) to establish a close symbiotic relationship opens new hypotheses on the pathogen association role in the induction of cancer. *M. hominis* infection dramatically upregulates the host inflammatory response to *T. vaginalis*. Hence, a marked chronic inflammatory state is a condition that predisposes to tumor transformation (Margarita et al., 2020).

Another novel promising trend is to create non-specific mutations into a variety of genes of *Mycoplasma* through DNA transformation and recombination. It was tested for creating knockout mutants ensuring that gene recombination is a successful approach for generating site-specific mutants and developing a new genetic system. This strategy will be a pioneering way to study pathogen-host interaction and pave the way to develop new genetically well-defined vaccine strains (Clampitt, 2021). Genome-scale models (GEMs) are a computational description of gene-protein reaction (GPR) associations for the whole metabolism of the target organism. Their reactions are ratio-based and mass-balanced, whereas, their formulation is based on experimentally gained gene annotation data. They help us through whole-cell analysis of an organism’s metabolic parameters. In addition, genome-scale metabolic models input external elements such as media constituents by simulating the metabolic fluxes. Then relate them to the bacterium growth, which can be described as biomass yield in the objective function of the model (Fang et al., 2020). The main computational approach that is applied to GEMs is Flux Balance Analysis (FBA) as a constraint-based model system that gives the expectation of metabolic fluxes through linear programming. It consists of a mathematical representation of the metabolic reactions (Nobile et al., 2021). More recently, Li et al. have explained the mechanisms of virulence attenuation using whole-genome sequencing and comparative genomic analysis of two *M. hyo* strains. The highly virulent *M. hyo* strain ES-2 has transformed to attenuated strain ES-2L with lower virulence after the *in vitro* serial passage of 200 times (Li et al., 2021).

Furthermore, proteomics techniques are frequently used. For instance, an immunoproteomics study on *M. bovis* has identified MbovP579 as a good diagnostic marker (Khan et al., 2016). A comparative secretome analysis on *M. bovis* virulent P1 and attenuated P150 strains has recognized a differential secretory protein of MbovP0145 as a potential diagnostic antigen (Zhang et al., 2021a). The *in silico* analysis of secretory lipoproteins revealed the apoptosis inducer MbovP280 of *M. bovis via* its interactive CRYAB (Zhao et al., 2021a). Above all, Multi-Omics Technology (proteomics, transcriptomics, metabolomics, etc.) by using over one omics technique would enable us to intensively and systematically understand mycoplasmas. Besides, the potential candidate targets for the development of diagnostic reagents, novel vaccines, and drugs can be discovered (Jores et al., 2020). Gaspari (Gaspari, 2021) has developed a genome-scale, constraint-based model, and metabolic modeling to determine the factors affecting the growth of *M. pneumoniae.* Whatsmore, she established a pioneering technique that allows the growth of *M. pneumoniae* on serum-free media.

## Eradication of Mycoplasmosis

### Eradication of CBPP in Many Parts of the World

CBPP has been completely eradicated from many parts of the world (OIE, 2019). For instance, in China the disease caused considerable economic losses to the cattle industry between the 1950s and 1970s. A potent vaccine, developed from a virulent strain of *Mmm* (Ben-1), was attenuated through multiple passages in rabbits. It had high immunogenicity and a remarkable protection efficacy (95-100%) in cattle for 28 months (Xin et al., 2012). Sheep were used for preparing this vaccine to increase the antigen yield and then in Tibetan sheep where it led to fewer adverse effects in domestic yaks and related species. Finally, the last CBPP case was recorded in 1989 and in 2008; OIE has announced China to be a CBPP-free country (Xin et al., 2012). More recently, this potent strain was trialled in Africa and reported to be as effective as T1/44 though the full data has not yet been available (Jores et al., 2020).

In Europe, where vaccination was prohibited for most of the 20th Century, slaughter of affected and in contact cattle has been the only method of control. This was largely successful, and it was believed that CBPP had been eradicated by the mid1960s. However, CBPP re-emerged two decades later in Portugal, Spain, France, and Italy but was finally eradicated following strict stamping out when the last case was recorded in Portugal in 1999 (Nicholas et al., 2008).

In Australia, the vaccination campaigns using attenuated vaccine strains (KH3J and T1/44) successfully reduced the number of cases. But the total eradication was achieved in 1973 only after applying strict animal movement measures and stamping out policy (Newton, 1992).

### Eradication of *M. bovis* in New Zealand

*M. bovis* infection was first reported in New Zealand in 2017. The Ministry for Primary Industries (MPI) took a brave action to eradicate *M. bovis* from NZ despite the presented difficulties in detecting and containing the movement of infected livestock. Decisively, it was achieved *via* culling of infected herds, besides, the NZ Government’s decision to eradicate *M. bovis* was unique since no other country has attempted it previously (Boyce et al., 2021). MPI has designed a new website that contains the latest updates and *M. bovis* situation reports, compensation, community events, and the national surveillance concerning eradication. Subsequently, the Chair of the Technical Advisory Group to MPI for the *M. bovis* program on 19 August 2021 has announced that currently there are only three active properties of *M. bovis*. Soon afterward, the eradication may be achievable. However, long-term surveillance will be required before freedom of infection can be declared (Controlling M. bovis in NZ 2021).

### *M. hyo* Eradication Program

More recently, Gulliksen and his co-workers have announced the eradication of *M. hyo* infections in the Norwegian pig population (Gulliksen et al., 2021). Their strategy was based on the implementation of numerous factors, such as well documented and effective eradication protocols, paving the way for designing accurate diagnostic tests, decreasing herd density, stopping the importation of live animals, besides, the loyalty of farmers and substantial efforts of veterinarians for rapid sampling and diagnosis. Following their path in this concern, many other mycoplasmas can be completely eradicated in the coming few years.

## Conclusions

Rampant *Mycoplasma* pathogens have caused great concern in recent years. Up to date, few intensive studies have been performed to investigate the recent clinical implications, virulence-related factors, and the reported non-specific host infection of different mycoplasmas as host-specific and pantropic pathogens. We summarized the most recent clinical implication in human and different animals species, virulence-related factors, common proteins incorporated in *Mycoplasma* infection, and the pivotal influence of the gene transfer process on *Mycoplasma* pathogens’ evolution. Furthermore, the immune response of *Mycoplasma* pathogens as unique antigens with limited metabolic capacities has a greater influence than others. Future perspectives with advances in the nanotechnology field had been shown as a new ingenious field to stop the growing threat of mycoplasmosis. Therefore, the ultimate but challenging goal is referring to study and explore using of nanoparticles in the *Mycoplasma* field as brilliant nanocarriers. Animal models also can assist us with superior outcomes for diagnosing and treating mycoplasmosis. Subsequently, it is vital to adopt modern technological methods to verify the mechanism of mycoplasmas infection. This will lay a foundation to create a new paradigm of diagnostics and therapeutic formulations and interventions. Finally, many scenarios for achieving mycoplasmas eradication in many parts of the world have succeeded that can be followed by others.

## Author Contributions

AD and AG conceived the project, reviewed the articles, and extracted the data. AD and SA wrote the manuscript. GZ, TZ, MQ, KD, ZH, MA, and IS reviewed the articles. All authors approved the submission of the manuscript.

## Funding

This work was supported by National Natural Science Foundation Projects (#31772745), the Key Research and Development Program of the Ningxia Hui Autonomous Region (# 2021BEF02028), and the China Agriculture Research System (Beef/yaks) of MOF and MARA (#CARS-37).

## Conflict of Interest

The authors declare that the research was conducted in the absence of any commercial or financial relationships that could be construed as a potential conflict of interest.

## Publisher’s Note

All claims expressed in this article are solely those of the authors and do not necessarily represent those of their affiliated organizations, or those of the publisher, the editors and the reviewers. Any product that may be evaluated in this article, or claim that may be made by its manufacturer, is not guaranteed or endorsed by the publisher.

## Glossary

**Table:**

M	Mycoplasma
U	Ureaplasma
MG	M. gallisepticum
Mmm	Mycoplasma mycoides subspecies mycoides
MS	M. synoviae
MM	M. meleagradis
Mcc	Mycoplasma capricolum subspecies capripneumoniae
Mmc	Mycoplasma mycoides subspecies capri
MI	M. iowae
M. hyo	M. hyopneumoniae
MDR	Multidrug resistance
CAP	Community-acquired pneumonia
HCC	Hepatocellular carcinoma
COPD	Chronic obstructive pulmonary disease
HGT	Horizontal gene transfer
ARGs	Antibiotic resistance genes
VED	Vaccine-enhanced disease
EpCAM	Epithelial cell adhesion molecule
MRMP	Macrolide-resistant M. pneumoniae
LAMPs	Lipid-associated membrane proteins
MEV	Multi-epitope vaccine
MALDI-TOF MS	Matrix-Assisted Laser Desorption/Ionization-Time Of Flight- mass spectrometry
PID	Pelvic inflammatory disease
NGU	Nongonococcal urethritis
ATM	Acute transverse myelitis
SPF	Specific-pathogen-free
CBPP	Contagious bovine pleuropneumonia
CCPP	Contagious caprine pleuropneumonia
BRD	Bovine respiratory disease
PARP-1	Poly-(ADP-ribose) Polymerase
PRDC	Porcine respiratory disease complex
CA	Contagious agalactia
MLST	Multilocus sequence typing
MLVA	Multilocus variable-number tandem repeat analysis
HM	Hemotropic mycoplasmas
PHs	Porcine hemoplasmas
M equi	M. equigenitalium
LAMP	Loop-mediated isothermal amplification assay
ANIs	Average nucleotide identity
LRT	Lower respiratory tract
Mhf	haemofelis
Mhc	M. hemocanis
CMhp	Candidatus M. haematoparvum
CMhm	Candidatus M. haemominutum
CMt	Candidatus M. turicensis
IKC	infectious keratoconjunctivitis
Th2	T helper-2 cells
PBVAs	Peri-bronchovascular areas
HMW	High molecular weight proteins
ILs	Inter Leukines
TGF-β1	Transforming growth factor beta-1
DCs	dendritic cells
RANTES	Regulated on Activation Normal T Cell Expressed and Secreted interleukine
TLR	Toll-like Receptor
TNF-α	tumor necrosis factor-alpha
NF-κB	Nuclear factor kappa B
ICAM-1	Endothelial cell adhesion molecules-1
AMs	Alveolar macrophages
NETs	Neutrophil extracellular traps
AMMY	anti-Mycoplasma mycoides subspecies mycoides
mAbs	monoclonal antibodies
BoLEC	Bovine lung epithelial cells
CPS	capsular polysaccharide
IHA	Immunohistological analysis
VCAM-1	vascular cell adhesion molecule-1
ICAM-1	Intercellular adhesion molecule-1
MMPs	Matrix Metalloproteases
MBA	Major band antigen
MnuA	major membrane nuclease A
PBMCs	Peripheral blood mononuclear cells
MNCs	Mononuclear cells
CFD	Complement factor D
TNFSF13	Tumor necrosis factor superfamily member 13
PCLS	Precision-cut lung slices
PD-1	Programmed cell death 1
PD-L1	Programmed cell death-ligand 1
LAG3	Lymphocyte activation gene 3
CTLA4	Cytotoxic T-lymphocyte- associated protein 4
MIB	Mycoplasma immunoglobulin binding
MIP	Mycoplasma immunoglobulin protease
ECM	Extracellular matrix
ROS	reactive oxygen species
PAMPs	Pathogen-associated molecular patterns
DAMPs	Danger-associated molecular patterns
GtsABC	Glycerol transporter system ATP-binding cassette
MALP-2	Macrophage activating lipopeptide-2
PARCELs	Palindromic Amphipathic Repeat Coding Elements
VSPs	Variable surface proteins
CARDS	Community-acquired respiratory distress syndrome
BBH	Bidirectional best hit approach
OCs	Open reading frame clusters
MBL	Mannose-binding lectins
MGEs	Mobile genetic elements
ICEs	Integrative and conjugative elements
CGIC	Colloidal gold-monoclonal antibody immunochromatography
SWCNT	Single-walled carbon nanotubes
LAMP	Loop-mediated isothermal amplification
LFB	Lateral flow biosensor assay
CRDC	Canine respiratory disease complex
MCDA	Multiple cross displacement amplification techniques
NPs	Nanoparticles
ZnO-NPs	Zinc oxide nanoparticles
NA-SERS	Nanorod array-surface enhanced Raman spectroscopy
AgNWs	Silver-nanowires
GEMs	Genome-scale models
Micro RNAs	miRNAs
GPR	Gene-protein reaction
FBA	Flux Balance Analysis
MPI- NZ	Ministry for Primary Industries in New Zealand.
