# Corrigendum: mycoplasmas as host pantropic and specific pathogens: clinical implications, gene transfer, virulence factors, and future perspectives

**DOI:** 10.3389/fcimb.2022.983282

**Published:** 2022-08-09

**Authors:** Ali Dawood, Samah Attia Algharib, Gang Zhao, Tingting Zhu, Mingpu Qi, Kong Delai, Zhiyu Hao, Marawan A. Marawan, Ihsanullah Shirani, Aizhen Guo

**Affiliations:** ^1^ The State Key Laboratory of Agricultural Microbiology, (HZAU), Wuhan, China; ^2^ College of Veterinary Medicine, Huazhong Agricultural University, Wuhan, China; ^3^ Department of Medicine and Infectious Diseases, Faculty of Veterinary Medicine, University of Sadat City, Sadat City, Egypt; ^4^ Hubei Hongshan Laboratory, Wuhan, China; ^5^ National Reference Laboratory of Veterinary Drug Residues (HZAU) and MAO Key Laboratory for Detection of Veterinary Drug Residues, HZAU, Wuhan, China; ^6^ Department of Clinical Pathology, Faculty of Veterinary Medicine, Benha University, Toukh, Egypt; ^7^ Hubei International Scientific and Technological Cooperation Base of Veterinary Epidemiology, Huazhong Agricultural University, Wuhan, China; ^8^ Infectious Diseases, Faculty of Veterinary Medicine, Benha University, Toukh, Egypt; ^9^ Para-Clinic Department, Faculty of Veterinary Medicine, Jalalabad, Afghanistan

**Keywords:** mycoplasmas, pantropic pathogens, virulence factors, clinical implications, gene transfer, vaccination

## Text correction

In the published article, there was an unclear sentence.

A correction has been made to **Clinical implications of mycoplasmas**, *Human infection*, Paragraph Number 4. This sentence previously stated:

“While in other countries (China, Japan, and Korea)”

The corrected sentence appears below:

“While in other countries and regions (China mainland, Japan, and Korea)”

The authors apologize for this error and state that this does not change the scientific conclusions of the article in any way. The original article has been updated.

## Publisher’s note

All claims expressed in this article are solely those of the authors and do not necessarily represent those of their affiliated organizations, or those of the publisher, the editors and the reviewers. Any product that may be evaluated in this article, or claim that may be made by its manufacturer, is not guaranteed or endorsed by the publisher.

